# Interpreting structure in sequence count data with differential expression analysis allowing for grades of membership

**DOI:** 10.1101/2023.03.03.531029

**Published:** 2023-03-06

**Authors:** Peter Carbonetto, Kaixuan Luo, Abhishek Sarkar, Anthony Hung, Karl Tayeb, Sebastian Pott, Matthew Stephens

**Affiliations:** 1 Department of Human Genetics, University of Chicago, Chicago, IL, USA.; 2 Research Computing Center, University of Chicago, Chicago, IL, USA.; 3 Department of Statistics, University of Chicago, Chicago, IL, USA.; 4 Section of Genetic Medicine, University of Chicago, Chicago, IL, USA.; 5 Committee on Genetics, Genomics and Systems Biology, University of Chicago, Chicago, IL, USA.; 6 Vesalius Therapeutics, Cambridge, MA, USA.

**Keywords:** gene expression, single cell, RNA-seq, ATAC-seq, differential expression analysis, dimensionality reduction, parts-based representations, matrix factorization, topic modeling

## Abstract

“Parts-based” representations of data, such as non-negative matrix factorization and topic modeling, have been used to identify structure from single-cell sequencing data sets, in particular structure that is not as well captured by clustering or other dimensionality reduction methods. However, interpreting the individual “parts” remains a challenge. To address this challenge, we extend methods for differential expression analysis by allowing the cells to have partial membership in multiple groups (or topics). We call this new approach *grade of membership differential expression* (GoM DE). We illustrate the benefits of GoM DE for annotating topics identified in several single-cell RNA-seq and ATAC-seq data sets.

## Background

A key methodological aim in single-cell genomics is to learn structure from single-cell sequencing data in a systematic, data-driven way [1–3]. Clustering [4–7] and dimensionality reduction techniques such as such as PCA [8–10], *t*-SNE [11] or UMAP [12] are commonly used for this aim. Despite the fact that many of these techniques have been applied “out-of-the-box” (with some caveats [13–18]), they have been remarkably successful in revealing and visualizing biologically interesting substructures from single-cell data [7, 19–29].

Another class of dimensionality reduction approaches that have been used to identify structure from single-cell data are what are sometimes called *parts-based representations*—these approaches include non-negative matrix factorization (NMF) [30–44] and topic modeling [45–56], which also have formal connections [48, 57, 58]. Parts-based representations share some of the features of both a clustering and a dimensionality reduction: on the one hand, they learn a lower dimensional representation of the cells; on the other hand, the individual dimensions (the “parts”) of the reduced representation can identify discrete clusters when they exist in the data [59, 60]. However, parts-based representations are more flexible than clustering—the dimensions can also capture other features such as continuously varying cell states.

In this paper, we investigate the question of how to interpret the individual dimensions of a parts-based representation learned by fitting a topic model. (In the topic model, the dimensions are also called “topics”.) For topics that assign observations to discrete clusters, one could apply a standard method for differential expression analysis [61, 62] to compare expression between topics, then annotate these topics by the genes that are differentially expressed. The question, therefore, is what to do with topics that do not assign observations to discrete clusters. To tackle this question, we extend models that compare expression between groups by allowing observations to have *partial membership in multiple groups.* This more flexible differential expression analysis is implemented by taking an existing model and modifying it to allow for partial memberships to the groups or topics. This modified model is a “grade of membership” model [63], so we call our new method *grade of membership differential expression* (GoM DE). The idea is that, by generalizing existing methods, we can continue to take advantage of existing elements of differential expression analysis, but now apply them to learn about different types of cell features beyond discrete cell populations.

We describe the GoM DE approach more formally in the next section. Then we evaluate the GoM DE approach in simulations, showing, in particular, that it recovers the same results as existing differential expression analysis methods when the cells can be grouped into discrete clusters. In case studies, we demonstrate how the GoM DE analysis analysis can be used to uncover and interpret a variety of cell features from single-cell RNA-seq and ATAC-seq data sets.

## Results

### Methods overview and illustration

We begin by giving a brief overview of the topic model, then we describe the new methods for annotating individual topics. To illustrate key concepts, we analyze a single-cell RNA-seq (scRNA-seq) data set obtained from peripheral blood mononuclear cells (PBMCs) [29] that has been used in several benchmarking studies [e.g., 4, 7, 8, 64, 65]. We refer to these data as the “PBMC data.”

#### Learning expression topics from single-cell RNA-seq data

The original aim of the topic model was to discover patterns from collections of text documents, in which text documents were represented as word counts [45, 50, 66–68]. By substituting genes for words and cells for documents, topic models can be used to learn a reduced representation of cells by their membership in multiple “topics” [47].

When applied to scRNA-seq data generated using UMIs, the topic model assumes a multinomial distribution of the RNA molecule counts in a cell,

(1) $$x_{i1},\ldots ,x_{im}\sim Multinomial\left(s_{i};\pi_{i1},\ldots ,\pi_{im}\right).$$

where $s_{i}=x_{i1}+\cdots +x_{im}$. That is, the number of RNA molecules $x_{ij}$ observed for gene j in cell i is a noisy observation of an underlying true expression level, $\pi_{ij}$[8, 69].

For n cells, the topic model is a *reduced representation* of the underlying expression,

(2) $$\Pi ={LF}^{T},$$

where Π,L,F are n×m,n×K,m×K matrices, respectively, with entries $\pi_{ij},\;l_{ik},\;f_{jk}$. Each cell is represented by its “grade of membership” in K topics, a vector of proportions $l_{i1},\ldots ,l_{iK}$, such that $l_{ik}\geq 0,\;\sum_{k=1}^{K}\;l_{ik}=1$, and each “expression topic” is represented by a vector of (relative) expression levels $f_{1k},\ldots ,f_{mk},\;f_{jk}\geq 0$ (these are also constrained to sum to 1, which ensures that the $\pi_{ij}$’s are multinomial probabilities). To efficiently fit the topic model to large single-cell data sets, we exploit the fact that the topic model is closely related to the Poisson NMF model [48].

The matrix L in (2), which contains the membership proportions for all cells and topics, can be visualized using a “Structure plot”. Structure plots have been used with great success in population genetics [e.g., 70–72], and, more recently, to visualize the topics learned from bulk and single-cell RNA-seq data [47].

**Figure 1:**
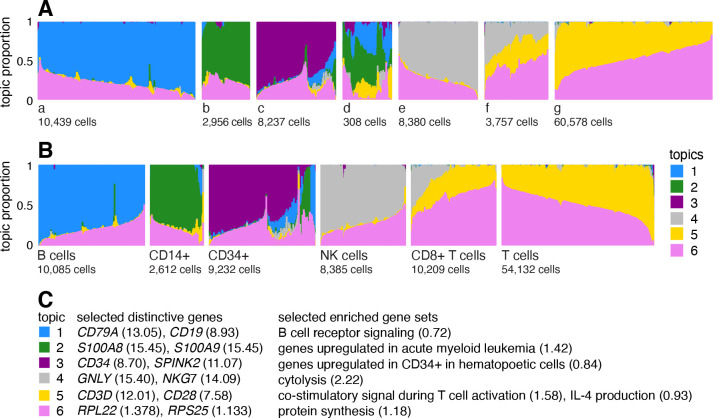
A and B give two views of the topic model fit to the PBMC data [29] (*n* = 94,655 cells, *K* = 6 topics) using Structure plots [70, 71]. Cells are arranged horizontally; bar heights correspond to cell topic proportions *l*_*ik*_. In A, the cells are arranged using the estimated topic proportions only. In B, the cells are grouped by the FACS labels. (The “T cells” label combines all sorted T cell populations other than CD8+ cytotoxic T cells.) In C, the topics are annotated by selected distictive genes from the GoM DE analysis (Fig. 2) and by selected enriched gene sets from the GSEA. Numbers in parentheses next to genes give posterior mean l.e. LFCs, and for gene sets they are enrichment coefficients. Note the groupings a–g in A are intended only to aid visualization. See also Additional file 1: Figure S4 for an alternative visualization.

A Structure plot visualizing the topic model fit to the PBMC data, with K=6 topics, is given in Fig. 1. In this data set, the cells have been “sorted” into different cell types which provides a cell labeling we can use to compare the topics. From the Structure plot, it is apparent that a subset of topics—topics 1, 2 and 3—correspond closely to sorted cell types (B cells, CD14+ monocytes, CD34+ cells), and, indeed, distinctive genes and enriched gene sets identified by the methods described below suggest these same cell types (Fig. 1C). Topics 4 and 5, on the other hand, are not confined to a single sorted cell type, and instead appear to capture biological processes common to T cells and natural killer (NK) cells. CD8+ cytotoxic T cells have characteristics of both NK cells and T cells—these are T cells that sometimes become “NK-like” [73]—and this is captured in the topic model by assigning membership to both topics. Topic 6 also captures continuous structure, but, unlike topics 4 and 5, it is present in almost all cells, and therefore its biological interpretation is not at all clear from the cell labeling. More generally, the topics, whether they capture largely discrete structure (topics 1–3) or more continuous structure (topics 4–6), can be thought of as a “soft” clustering [47].

#### Learning chromatin accessibility topics from single-cell ATAC-seq data

For single-cell ATAC-seq data, $x_{ij}$ is the number of DNA fragments mapping to peak j in cell i. Although $x_{ij}$ can take values 0,1, or 2, it is common to “binarize” the accessibility data [e.g., 19, 74–76], meaning that $x_{ij}=1$ when at least one fragment in cell i maps to region j and $x_{ij}=0$ otherwise.

The topic model for analyzing single-cell ATAC-seq data was first suggested in [49]. Although binarized chromatin accessibility values are most naturally modeled as binomial random variables, the topic model can be justified on the grounds that the multinomial model (1) is a good approximation to the binomial when ones are infrequent and the sample size is large.

#### Differential expression analysis allowing for grades of membership

Having learned the topics, our aim now is to identify genes that are distinctive to each topic. In the simplest case, the topic is a distinct or nearly distinct cluster of cells, such as topic 1 or 2 in Fig. 1.

In the following, we describe methods for analyzing *differences in expression*, but they can also be understood as methods for analyzing *differences in chromatin accessibility.* Therefore, “expression,” “expressed” and “gene” in the descriptions below may be substituted with “accessibility”, “accessible” and “peak” (or “region”).

Consider a single gene, j. Provided unmodeled sources of variation are negligible relative to measurement error, a simple Poisson model of expression should suffice:

(3) $$x_{ij}\sim Poisson\left(s_{i}\theta_{ij}\right).$$

In this model, the $\theta_{ij}$ for gene j in cell i is controlled by the cell’s membership in the cluster: when cell i belongs to the cluster, $\theta_{ij}=p_{j1}$; otherwise, $\theta_{ij}=p_{j2}$. Under this model, differential expression (DE) analysis proceeds by estimating the log-fold change (LFC) in expression for each gene j,

(4) $$LFC(j)={log}_{2}\frac{p_{j1}}{p_{j2}}.$$

Although simple, this Poisson model forms the basis for many DE analysis methods [e.g., 77], including edgeR [78, 79] and DESeq [80, 81].

We now modify the Poisson model (3) in a simple way to analyze differential expression among topics. In a clustering, each cell belongs to a single cluster, whereas in the topic model, cells have *grades of membership* to the clusters [63], in which $l_{ik}$ is the membership proportion for cluster or topic k. Similarly, we extend the Poisson model (3) to allow for partial membership in the K topics:

(5) $$\begin{array}{rr} & x_{ij}\sim Poisson\left(s_{i}\theta_{ij}\right)\\ & \theta_{ij}=\sum_{k=1}^{K}\;\;l_{ik}p_{jk}, \end{array}$$

in which the topic proportions $l_{ik}$ are treated as known, and the unknowns $p_{j1},\ldots ,p_{jK}$ represent relative expression levels. Note that $p_{jk}$ will be similar to, but not the same as, $f_{jk}$ in the topic model because the DE analysis is a gene-by-gene analysis, whereas the topic model considers all genes at once. The standard Poisson model (3) is recovered as a special case of (5) when K=2 and all topic proportions $l_{ik}$ are 0 or 1.

Recall, our aim is to identify genes that are *distinctive* to each topic. To this end, we estimate the *least extreme LFC* (l.e. LFC), which we define as

(6) $$LFC_{k}^{\text{l.e.}\;}(j):={\text{LFC}}_{k,l}(j)\;\;\;\;\;\;\;\;\;\text{such}\;\text{that}\;l={\mathrm{argmin}}_{l^{\prime }\neq k}\left|LFC_{k,l^{\prime }}(j)\right|,$$

in which ${LFC}_{k,l}(j)$ is the *pairwise* LFC,

(7) $${\mathrm{LFC}}_{k,l}\left(j\right):=\log_{2}\frac{p_{jk}}{p_{jl}}.$$

By this definition, a “distinctive gene” is one in which its expression is significantly different from its expression in *all other topics.* (Note the l.e. LFC reduces to the standard LFC (4) when K=2.) We then annotate topics by the distinctive genes. The computation of posterior l.e. LFCs estimates and related posterior statistics is described in Methods.

#### Illustration of GoM DE analysis in PBMC data set

**Figure 2:**
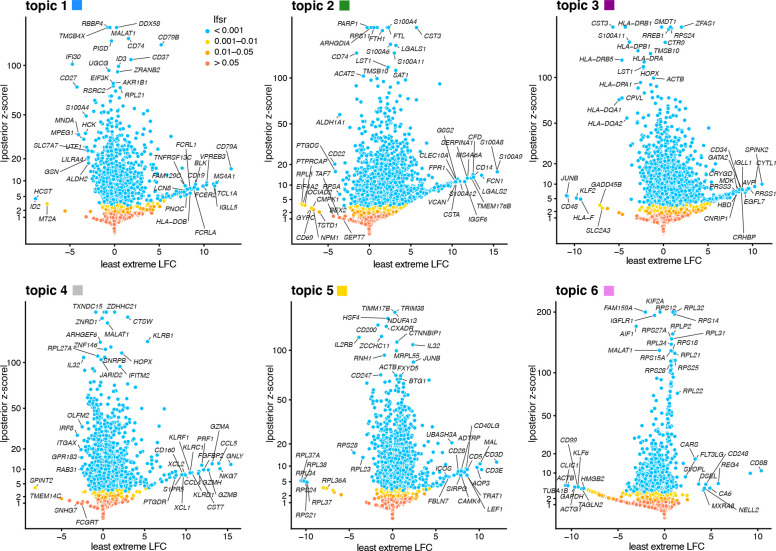
GoM DE analysis of the PBMC data using the topic model shown in Fig. 1. The volcano plots show moderated posterior mean estimates of the least extreme LFC vs. posterior *z*-score magnitudes for 17,055 genes. Genes are colored according to the estimated local false sign rate (*lfsr*) [82]. A small number of genes with extreme posterior *z*-scores are shown with smaller posterior *z*-scores so that they fit within the y-axis range. See also the detailed GoM DE results (Additional file 4: Table S1), interactive volcano plots (Additional file 2) and detailed GSEA results (Additional file 5: Table S3, S4).

To illustrate, we applied the GoM DE analysis to the topic model shown in Fig. 1, and visualized the results in “volcano plots” (Fig. 2). We then used the GoM DE results (Additional file 4: Table S1) to perform gene set enrichment analysis (Additional file 5: Tables S3, S4).

For the topics that closely corresponded to cell types, the GoM DE analysis identified genes and gene sets reflecting these cell types, as expected. For example, topic 1 corresponds to FACS B cells, and is characterized by overexpression of *CD79A* (posterior mean l.e. LFC = 13.05) and enrichment of B cell receptor signaling genes (enrichment coefficient = 0.72). Topic 2 corresponds to myeloid cells and is characterized by overexpression of *S100A9* (l.e. LFC = 15.45) and enrichment of genes down-regulated in hematopoietic stem cells (enrichment coefficient = 0.90).

**Figure 3:**
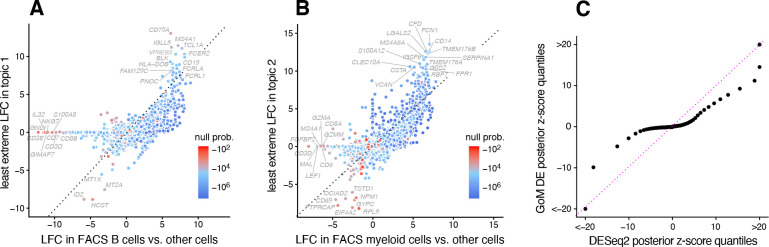
GoM DE analysis vs. DE analysis in FACS cell populations. Panels A and B compare differential expression in topics 1 and 2 (Fig. 1) with their closely corresponding FACS cell populations. Genes are only shown if the posterior *z*-score was greater than 2 in magnitude in at least one of the DE analyses. Genes are colored by the binomial null-model probability. The Q-Q plot (C) compares the overall distribution of posterior *z*-scores for B cells and myeloid cells (X-axis) and for topics 1 and 2 (Y-axis). For better visualization of quantiles near zero, posterior *z*-scores larger than 20 in magnitude are shown as 20 or −20. Analysis of different expression among the 6 FACS cell populations was performed by applying DESeq2 [81, 84] to the PBMC data.

The close correspondence between topics 1 and 2 and FACS cell types (B cells, myeloid cells) provides an opportunity to contrast the GoM DE analysis with a standard DE analysis of the FACS cell types (Fig. 3). This is not a perfect comparison because the topics and FACS cell populations are not exactly the same, but the LFC estimates correlate well (Fig. 3A, B). This comparison serves to illustrate to two key differences:

1. Many more l.e. LFCs are driven toward zero in theGoM DE analysis (Fig. 3C), so the l.e. LFCs more effectively draw attention to “distinctive genes” (Fig. 3A, B). This includes genes that are *distinctively underexpressed* such as *ID2* in B cells [83].
2. The GoM DE analysis yields much larger LFC estimates of the cell-type-specific genes. This is because the topic model isolates the biological processes related to cell type (topics 1 and 2) while removing background biological processes (topic 6) that do not relate to cell type.

Other topics capture more continuous structure, such as topics 4 and 5 (Fig. 1). Although the GoM DE analysis of these topics is not comparable to a standard DE analysis, many of the the distictive genes and gene sets suggest NK and T cells, which are precisely the FACS-labeled cells with greatest membership to these topics: for example, for topic 4, overexpression of *NKG7* (posterior mean l.e. LFC = 14.09), enrichment of cytolysis genes (enrichment coefficient = 2.22); for topic 5, overexpression of *CD3D* (l.e. LFC = 12.01), enrichment co-stimulatory signaling during T-cell activation (enrichment coefficient = 1.58).

Topic 6 captures continuous structure and is present in almost all cells, so knowledge of the FACS cell types is not helpful for understanding this topic. Still, the GoM DE results for topic 6 show a striking enrichment of ribosome-associated genes (Fig. 2, Additional file 5: Tables S3, S4). (These ribosomal protein genes account for a large fraction of the total expression in the PBMC data [5].) This ability to annotate distinctly non-discrete structure is a distinguishing feature of the grade-of-membership approach, and below we will show more examples where this feature contributes to understanding of the cell populations.

### Evaluation of DE analysis methods using simulated data

Having illustrated the features of this approach, we now evaluate the methods more systematically in simulated expression data sets. We began our evaluation by first considering the case of two groups in which there is no partial membership to these groups; that is, when the cells can be clearly separated into two cell types. The GoM DE analysis should accommodate this special case, and should compare well with existing DE analysis methods. We compared with DESeq2 [81] and MAST [85], both popular methods that have been shown to be competitive in benchmarking studies [61, 62, 86] (and are included in Seurat [25]).

**Figure 4:**
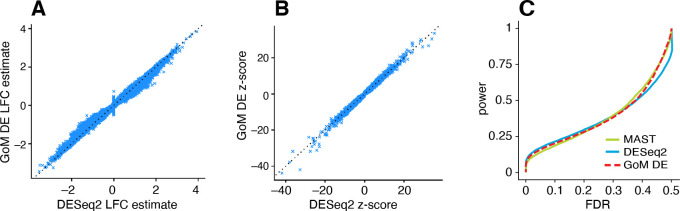
Evaluation of DE analysis methods in single-cell expression data sets in which cells were simulated from two groups without partial membership to these groups. Panels A and B compare posterior mean LFC estimates and posterior *z*-scores returned by DESeq2 [81] and the GoM DE analysis. Each plot shows 200,000 points for 10,000 genes × 20 simulated data sets. Panel C summarizes accuracy in identifying differentially expressed genes in all simulated data sets; it plots power and false discovery rates (FDR) for the three methods compared as the *p*-value (MAST [85]), *s*-value (DESeq2) or *lfsr* threshold (GoM DE) is varied from 0 to 1. Power and FDR are calculated from the number of true positives (TP), false positives (FP), true negatives (TN) and false negatives (FN) as FDR = FP/(TP + FP) and power = TP/(TP + FN). For additional results, see Additional file 1: Figures S6, S7.

To compare the ability of these methods to discover differentially expressed genes, we simulated RNA molecule count data for 10,000 genes and 200 cells in which 98% of cells were attributed to a single topic (with membership proportions of 99% or greater). Half of the simulated genes had different expression levels in the two topics. Molecule counts were simulated using a Poisson measurement model so that variation in expression across cells was due to either measurement error or true differences in expression levels between the two groups. For all DE analyses, we took group/topic assignments to be known so that incorrect assignment of cells to topics was not a source of error. Other aspects of the simulations were chosen to emulate molecule count data from scRNA-seq studies (see Methods for full simulation details). We repeated the simulations 20 times, and summarized the results of the DE analyses in Fig. 4 (see also Additional file 1: Figures S6, S7).

DESeq2 and the GoM DE analysis have several common features—both are based on a Poisson model, and both use adaptive shrinkage [82, 84] to improve accuracy of the LFC estimates and test statistics—so we expected the GoM DE analysis to closely replicate DESeq2 in these simulations. Indeed, both methods produced nearly identical posterior mean LFC estimates, posterior *z*-scores (Fig. 4A, B) and *s*-values (Additional file 1: Figure S7), and achieved very similar performance (Fig. 4C). Although DESeq2 additionally estimates an overdispersion level for each gene, in these simulations DESeq2 correctly determined that the level of overdispersion was small for genes with large expression differences, which explains the strong similarity of the LFC estimates and posterior *z*-scores. MAST, owing to an approach that is very different from DESeq2 and the GoM DE analysis, yielded estimates that were less similar (Additional file 1, Figure S7), yet achieved comparable performance (Fig. 4C).

Next we evaluated the GoM DE analysis methods in data sets in which the cells had varying degrees of membership to multiple topics. Since existing DE methods cannot handle the situation in which there are partial memberships to groups, we mainly sought to verify that the method behaves as expected in the ideal setting when data sets are simulated from the topic model (2). To provide some baseline for comparison, we also applied the method of Dey *et al* [47], which is not strictly a DE analysis method, but does provide a ranking of genes by their “distinctiveness” in each topic. This ranking is based on a simple Kullback-Leibler (K-L) divergence measure; large K-L divergences should signal large differences in expression, as well as high overall levels of expression, so large K-L divergences should correspond to small DE *p*-values. Since the K-L divergence is not a signed measure, we omitted tests for negative expression differences from the evaluations, which was roughly half of the total number of possible tests for differential expression.

We performed 20 simulations with K=2 topics and another 20 simulations with K=6 topics. To simplify evaluation, all genes either had the same rate of expression in all topics, or the rate was different in exactly one topic. As a result, the total number of expression differences in each data set was roughly the same regardless of the number of simulated topics. Other aspects of the simulations were kept the same as the first set of simulations; see Methods for details. Similar to before, we took the topic proportions to be known so that mis-estimation of the topic proportions would not be source of error in the GoM DE analysis and in calculation of the K-L divergence scores.

**Figure 5:**
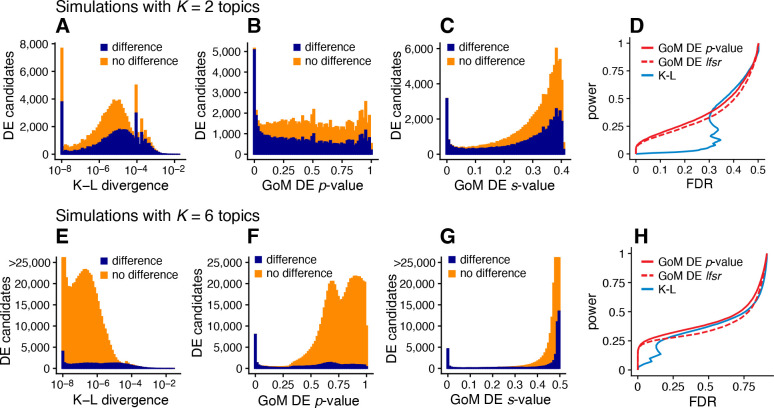
Evaluation of methods for identifying expression differences in single-cell expression data sets in which cells were simulated with partial membership to 2 topics (A–D) or 6 topics (E–H). Methods compared are the Kullback-Leibler (K-L) divergence score of [47] and GoM DE with adaptive shrinkage (*s*-values, *lfsr*) and without (*p*-values). The left-most panels (A, E) show the distribution of K-L divergence scores for all candidate expression differences (approximately half of 10,000 genes × 2 or 6 topics × 20 simulated data sets), shown separately for true expression differences (dark blue) and non-differences (orange). K-L divergence scores smaller than 10^−8^ are plotted as 10^−8^. Similarly, plots B, C, F, G show the distribution of GoM DE *p*-values or *s*-values with or without adaptive shrinkage, separately among differences and non-differences. Panels D, H summarize performance in identifying expression differences; it shows power and FDR as the GoM DE *p*-value or *lfsr* are varied from 0 to 1, or as the K-L divergence score is varied from large to small. Note that in E and G some bar heights are actually larger than 25,000 but are cut off at 25,000 for better visualization of shorter bars.

The largest K-L divergence scores in the simulated data sets reliably recovered true expression differences (Fig. 5A, E). Therefore, the K-L divergence scores achieved good *true positives rates* (*i.e.*, good power) at low *false positive rates*, FPR=FP/(TN+FP) (see Fig. 4 for notation). However, for DE analysis a more relevant performance measure is the *false discovery rate*, FDR=FP/(TP+FP). Because the K-L divergence score does not fully account for uncertainty in the unknown gene expression levels, many genes with no expression differences among topics were also highly ranked, leading to poor FDR control (Fig. 5D, H). By contrast, the GoM DE analysis better accounted for uncertainty in the unknown expression levels. The GoM DE analysis also more accurately recovered true expression differences at small *p*-values or *s*-values (Fig. 5B, C, F, G), and therefore obtained much lower false discovery rates at corresponding levels of power (Fig. 5D, H). Comparing the GoM DE analysis with and without adaptive shrinkage, the adaptive shrinkage did not necessarily lead to better performance (Fig. 5D, H), but did provide more directly interpretable measures of significance (*s*-values or local false sign rates) by shrinking the LFC estimates, and adapting the rate of shrinkage to the data; for example, the expression differences were shrunk more strongly in the K=6 data sets, correctly reflecting the much smaller proportion of true expression differences (compare Fig. 5C and G).

### Case study: scRNA-seq epithelial airway data from Montoro *et al*, 2018

We reanalyzed scRNA-seq data for n=7,193 single cells that were sampled from the tracheal epithelium in wild-type mice [87]. The original analysis [87] used a combination of methods, including *t*-SNE, community detection [88], diffusion maps [89], and partitioning around medoids (PAM) to identify 7 epithelial cell types: abundant basal and secretory (club) cells; rare, specialized epithelial cell types, including ciliated, neuroendocrine and tuft cells; a novel subpopulation of “ionocytes”; and a novel basal-to-club transitional cell type, “hillocks”. Although not large in comparison to other modern single-cell data sets, this data set is challenging to analyze, with complex structure, and a mixture of abundant and rare cell types. In contrast to the PBMC data set, there are no existing cell annotations to interpret the topics, so we must rely on inferences made from the expression data alone to make sense of the results.

**Figure 6:**
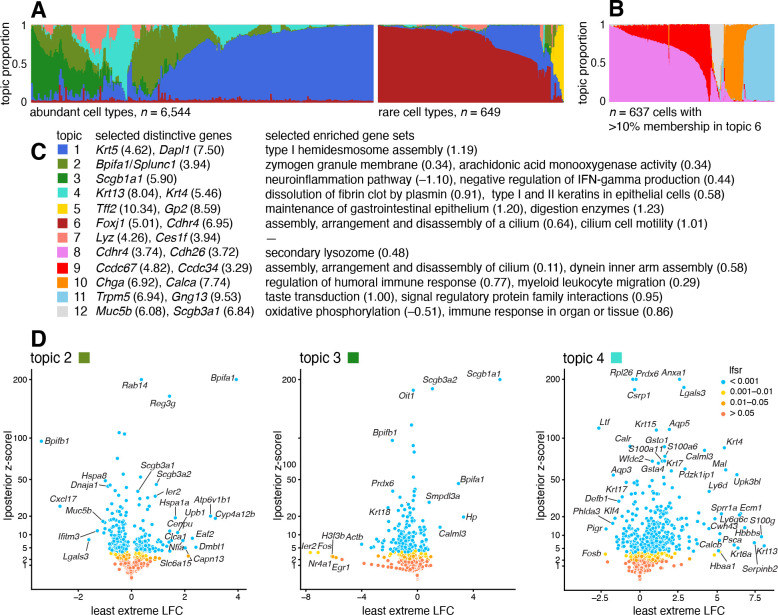
Structure in mouse epithelial airway data (*n* = 7,193 cells [87]) inferred from topic modeling (A, B), and GoM DE analysis (D) of selected topics using the topic proportions **L** shown in A. In C, the topics are annotated by selected distinctive genes (numbers in parentheses are posterior mean l.e. LFCs) and selected enriched gene sets (numbers in parentheses are posterior mean estimates of the enrichment coefficients). In A, to better visualize the rare cell types, the cells are divided into two groups, “abundant and rare,” based on the estimated topic proportions, and the “abundant” cells are subsampled to produce the Structure plot. The Structure plot in B is obtained by fitting another topic model, with *K* = 5 topics, to rare epithelial cell types (defined as the subset of 637 cells *i* with at least 10% membership to topic 6). The volcano plots show moderated posterior estimates of least extreme LFC vs. posterior *z*-score magnitudes for 18,388 genes. A small number of genes with extreme posterior *z*-scores are shown with smaller posterior *z*-scores so that they fit within the Y-axis range. See also the interactive volcano plots (Additional file 3: S9), GoM DE results for all topics (Additional file 4: Table S2, Additional file 1: Figures S9; S10), and the GSEA results (Additional file 5: Tables S5, S6).

The topic model fit to the UMI counts with K=7 topics is summarized in Fig. 6A and the results of the GoM DE analysis and subsequent GSEA are summarized in Fig. 6. Although we do not have cell labels to compare with, distinctive genes emerging from the GoM DE analysis help connect some of the topics to known cell types. For example, the most abundant topics correspond well with predominant epithelial cell types in the lung: topic 1 shows strong overexpression of basal cell marker gene *Krt5* [90] (posterior mean l.e. LFC = 4.62); distinctive genes in topics 2 and 3 include key secretory genes in club cells such as *Bpifa1*/*Splunc1* [91] (l.e. LFC = 4.93) and *Scgb1a1* [92] (l.e. LFC = 5.90).

The “hillock” transitional cells, which were originally identified via a diffusion maps analysis [87], emerge as a single topic (topic 4, cyan), with *Krt13* (l.e. LFC = 8.04) and *Krt4* (l.e. LFC = 5.46) being among the most distinctive genes (Fig. 6C). The transitional nature of these cells is evoked by their mixed membership; only 237 cells have >90% membership to this topic.

Other less abundant epithelial cell types emerge as separate topics once a topic model (with *K* = 5 topics) is fit separately to the subpopulation of these rare cell types (Fig. 6B). These topics recover ciliated (topics 8, 9; *Ccdc153*, posterior l.e. LFC = 5.39), neuroendocrine (topic 10; *Chga*, l.e. LFC = 6.92) and tuft cells (topic 11; *Trpm5*, l.e. LFC = 6.94). Note that *Foxi1+* ionocytes were previously identified as a novel cell type from a small cluster of 26 cells [87], but our analysis failed to distinguish this very rare cell type from neuroendocrine cells (Additional file 1: Figures S8, S11).

The topics also capture biologically relevant *continuous substructure* in club cells (topics 2 and 3) and ciliated cells (topics 8 and 9) that was not discovered in the original analysis [87]. This continuous substructure may be reflective of finer scale cell differentiation or specialization of function. In particular, we interpret topic 3 as capturing “canonical” or “mature” (*Scgb1a1+*, l.e. LFC = 5.90) club cells [91], with negative regulation of inflammation, whereas cells with greater membership to topic 2 are “clublike” (*Bpifa1*/*Splunc1+*, l.e. LFC = 3.94) [90, 92]. Topic 9, similarly, appears to represent “canonical” ciliated cells, featuring upregulated genes such as such as *Ccdc67*/*Deup1* (l.e. LFC = 4.82) and *Ccdc34* (3.29) [90, 93, 94], and enrichment of Gene Ontology terms [95] such as cilium organization (GO:0044782) and axonemal dynein inner arm assembly (GO:0036159).

In summary, taking a topic-model-based approach we identified and annotated well-characterized cell types such as basal cells, as well less distinct but potentially interesting substructures such as “Hillock” cells and club cell subtypes.

### Case study: Mouse sci-ATAC-seq Atlas data from Cusanovich *et al* (2018)

**Figure 7:**
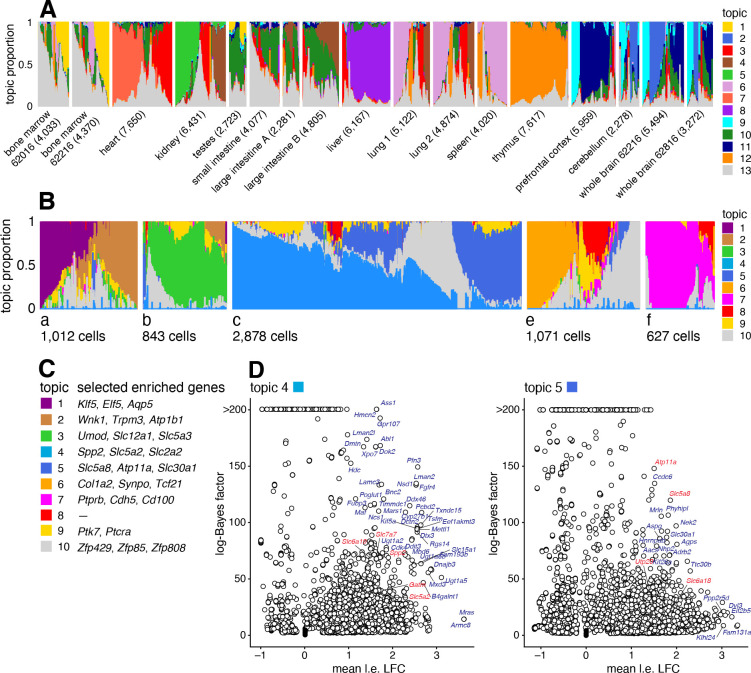
(A) Structure in Mouse Atlas sci-ATAC-seq data (*n* = 81,173) inferred from topic modeling, with *K* = 13 topics; (B) topic model fit to kidney cells (*n* = 6,431) with *K* = 10 topics; (C, D) gene-based enrichment analysis of differentially accessible peaks for the kidney cell topics shown in B, in which peaks are linked to genes using Cicero [96]. In A, the cells are grouped by tissue, and replicates (for bone marrow, large intensine, lung and whole brain) are shown as separate tissues. Numbers in parentheses next to each tissue give the number of cells in that tissue. In D, marker genes for S1 (topic 4) and S3 (topic 5) proximal epithelial tubular cells are highlighted in red (marker genes are listed in Table 1 of [97]). “Mean l.e. LFC” is the average least extreme LFC among all peaks connected to the gene, restricted to l.e. LFCs with *lfsr* < 0.05. Log-Bayes factors greater than 200 are shown as 200 in the volcano plots. (See Additional file 1: Figure S14 and Additional file 6: Table S7 for more gene enrichment results.) In B, the cells are subdivided into 5 groups (a–f) for improved visualization only. See also Additional file 1: Figure S13 which compares the topics in A to the Cusanovich *et al* [98] cell-type predictions based on clustering.

We reanalyzed data from the Mouse sci-ATAC-seq Atlas [98], comprising 81,173 single cells in 13 tissues. First, to provide an overview of the primary structure in the whole dataset, we fit a topic model with K=13 topics to these data (Fig. 7A). Several different tissues are clearly distinguished by different topics, and the replicates have a similar distribution of topics. Additionally, the topics correspond closely to the clusters identified in [98] (Additional file 1: Figure S12).

Next we performed a more detailed analysis of just the kidney (6,431 cells), fitting a topic model with K=10 to just these cells. We focussed on the kidney cells because, as noted previously [98, 99], both expression and chromatin accessibility vary in relation to the spatial organization of the renal tubular cells, and we predicted that this spatial structure could be better captured by topics than by traditional cluster methods. To interpret these topics obtained from chromatin accessibility data, we first used the GoM DE analysis to identify differentially accessible peaks for each topic, and then used “co-accessibility” as predicted by Cicero [96, 98] to connect genes to peaks representing distal regulated sites. Finally, we performed a simple enrichment analysis to identify the “distinctive genes” for each topic, which we defined as the genes with many distal regulatory sites that were differentially accessible.

The results are shown in Fig. 7. Many of the distinctive genes (Fig. 7, Additional file 1: Figure S14, Additional file 6: Table S7) clearly relate topics to known kidney cell types. For example, topic 1 is enriched for genes *Klf5*, *Elf5*, which relate to the collecting duct [99, 100]); topic 3 is enriched for genes *Umod*, *Slc12a1* associated with the loop of Henle [99, 101]); and topics 2, 6, 8 are respectively enriched for genes related to the distal convoluted tubule (*Wnk1*), podocytes (*Col1a2*) and glomerular endothelial cells (*Ptprb*).

Most interestingly, spatial organization of the proximal tubule is captured by two topics; topic 4 is enriched for *Slc5a2* (also known as *Sglt2*) and *Slc2a2* (also known as *Glut2*), associated with the S1 segment of the proximal tube [97, 102, 103]) and topic 5 is enriched for *Slc5a8* (*Smct1*) and *Atp11a*, related to the S3 segment [97, 104]). This result illustrates the ability of the topic model to capture continuous variation in membership of two (somewhat complementary) processes, which traditional clustering methods are not designed to do.

### Case study: chromatin accessibility profiles of the hematopoietic system from Buenrostro *et al* (2018)

**Figure 8:**
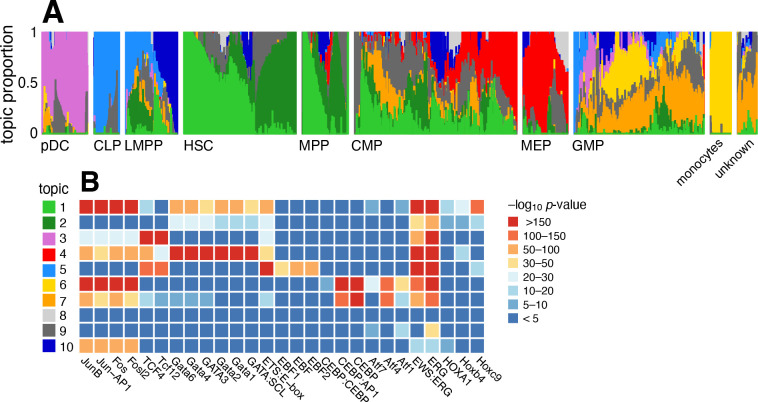
Structure in human hematopoietic system data (*n* = 2,034 cells [105]) inferred from the topic model with *K* = 10 topics (A), and HOMER motif enrichment analysis [106] applied to the results of the GoM DE analysis (B). In the Structure plot, the cells are grouped by FACS classification, as well as an unknown population from human bone marrow [105]. Panel B shows HOMER enrichment results for selected motifs (for the full results, see Additional file 7: Table S8). Acronyms used: common lymphoid progenitor (CLP); common myeloid progenitor (CMP); granulocyte-macrophage progenitor (GMP); hematopoietic stem cell (HSC); lymphoid-primed multipotent progenitor (LMPP); megakaryocytic-erythroid progenitor (MEP); multi-potent progenitor (MPP); plasmacytoid dendritic cells (pDC).

Buenrostro *et al* [105] studied 2,034 single-cell ATAC-seq profiles of 10 cell populations isolated by FACS to characterize regulation of the human hematopoietic system. Both PCA and *t*-SNE show, visually, the expected structure into the main developmental branches (Fig. 2 in [105]). However, neither PCA nor *t*-SNE isolates these branches as *individual dimensions* of the embedding. Identifying these branches may allow for more precise characterization of the underlying regulatory patterns. Here, by fitting a topic model to the data, the main developmental branches are identified as individual topics (Fig. 8A): topic 3, pDC; topic 4, erythroid (MEP); topic 5, lymphoid (CLP); and topic 6, myeloid (GMP and monocytes). Another topic captures the cells at the top of the developmental path (topic 1; HSC and MPP). Other cells at intermediate points in the developmental trajectory, such as CMP, GMP and LMPP cells, are more heterogeneous, and this is reflected by their high variation in topic membership.

To better interpret the regulatory patterns behind each topic, we identified transcription factor (TF) motifs that were enriched for differentially accessible regions in each topic (Fig. 8B, Additional file 7: Table S8). Many of the top TF motifs (as ranked by HOMER *p*-value [106]) point toward regulation of main developmental trajectories, such as EBF motifs in topic 5 (lymphoid), CEBP motifs in topics 6 and 7 (myeloid), and Hox motifs in topic 1 (HSC and MPP cells). A few topics (topics 8–10) are much less abundant and do not align well with the FACS cell types, and their motif enrichment results were correspondingly more difficult to interpret.

A complication that arose in analyzing these data, which was also noted in [105], is that the cells were obtained from different sources, and this shows up as systematic variation in the chromatin accessibility; this batch effect is captured by topics 1 and 2 in HSC and MPP cells, and, to a lesser extent, in CMP and LMPP cells (Additional file 1: Figure S15). Topic 1 is enriched for Jun and Fos TF motifs, similar to what was found in [105]. We return to this complication in the Discussion.

## Discussion

The GoM DE analysis is part of a *topic-model-based pipeline* for analysis of single-cell RNA-seq [47] or ATAC-seq data [49]. It involves the following steps: (1) fit a topic model to the data; (2) visualize the structure inferred by the topic model; (3) run the GoM DE analysis with the estimated topics; and, optionally, (4) perform other downstream analyses using the results of the GoM DE analysis, e.g., gene set enrichment analysis (for RNA-seq data) or motif enrichment analysis (for ATAC-seq data). Unlike most analysis pipelines for clustering and dimensionality reduction (e.g., [4, 19, 23, 26, 27]) the topic-model-based pipeline is directly applied to the “raw” count data, and therefore does not require an initial step to transform and normalize the data which can lead to downstream issues in the statistical analysis [8, 107–109]. We presented several case studies illustrating the use of the topic-model-based pipeline to analyze single-cell RNAseq and ATAC-seq data sets. From these case studies, we have drawn a few lessons on some of the practical challenges that can arise in applying topic modeling approaches to single-cell data, and we share these lessons here. (See also [47, 49] for related discussion.)

One practical question is how to choose *K*, the number of topics. Instead of thinking of *K* as a tuning parameter with a single “correct” value, we follow [47] and recognize the advantages of learning topics at multiple settings of *K*; multiple *K*’s can reveal structure at different levels of granularity. We have found that it is often helpful to start with smaller *K* to elucidate the less granular structure, which is also typically easier to interpret, then rerun the topic modeling with larger *K* to identify finer structure.

Donor or other technical effects in the single-cell RNA-seq or ATAC-seq data, whether they are known or unknown, can complicate the analysis and interpretation of the topics if these effects are large. In the Mouse sci-ATAC-seq Atlas data, for example, the replicate effects appeared to be small (Fig. 7), so it seemed reasonable to ignore them. By contrast, in the human hematopoietic system data, the donor effects were large (Fig. 8), and indeed these donor effects were reflected in some of the topics. How to deal with nonignorable donor or batch effects in a topic modeling analysis remains an open question, and the best approach may depend on the particulars of the data set.

For modeling UMI counts, an open question is whether the Poisson or multinomial model (1) is suffcient, or whether more flexible models are needed. (This question was investigated in [69] for single-gene models, but not for multi-gene models.) Alternative models such as the negative binomial [110], which can capture additional random variation (“overdispersion”) in underlying expression or measurement error, may result in more robust estimation of the topics.

In single-cell ATAC-seq data, the GoM DE analysis identifies differentially accessible peaks or regions. Usually these peak-level results need to be translated into biological units that are more useful for annotating the topics (genes, gene sets, transcription factors). In the analysis of the hematopoietic system single-cell ATAC-seq data, we used HOMER [106] to identify TF motifs enriched for differentially accessible peaks in each topic. In the analysis of the Mouse sciATAC-seq Atlas data, we identified genes enriched for differentially accessible distal regularity sites in each topic. Clearly, the quality of the gene enrichment results will depend on our ability to accurately associate peaks with genes. For this, we used the scores computed in [98] using Cicero [96]. However, there are now several alternative approaches that may be preferred [19, 27, 28, 111–114], and in principle any of these approaches could be combined with the peak-level GoM DE results to identify relevant genes.

## Conclusions

To summarize, we have described a new method that aids in annotating and interpretating the “parts” of cells learned by fitting a topic model to scRNA-seq data or single-cell ATAC-seq data. Our method, GoM DE (differential expression analysis allowing for grades of membership), can be viewed as an extension of existing differential expression methods that allows for mixed membership to multiple groups or topics.

## Methods

### Differential expression analysis allowing for grades of membership

#### Derivation of GoM DE model

In the “Methods overview,” we motivated the GoM DE model (3) as extending a basic Poisson model expression to allow for partial membership to K groups or topics. The GoM DE model can also be motivated from an approximation to the topic model. Recall, the topic model is a multinomial model (1) in which the multinomial probabilities $\pi_{ij}$ are given by affine combinations of the expression levels $f_{jk}$ in the K top $ics,\pi_{ij}=\sum_{k=1}^{K}\;l_{ik}f_{jk}$. The non-negativity constraints $l_{ik}\geq 0,\;f_{jk}\geq 0$ and sum-to-one constraints $\sum_{k=1}^{K}\;l_{ik}=1,\;\sum_{j=1}^{p}\;f_{jk}=1$ ensure that the $\pi_{ij}$ ‘s are multinomial probabilities. From a basic identity relating the multinomial and Poisson distributions [115, 116], the multinomial likelihood for the topic model can be replaced with a likelihood formed by a simple product of independent Poissons; that is,

(8) $$\mathrm{Mulinomial}\left(x_{i};s_{i},\pi_{i}\right)\propto \prod_{j=1}^{m}\;\mathrm{Pois}\left(x_{ij};s_{i}\pi_{ij}\right),$$

where $x_{i}=\left(x_{i1},\ldots ,x_{im}\right)$ and $\pi_{i}=\left(\pi_{i1},\ldots ,\pi_{im}\right)$. The approximation then comes from no longer requiring the $\pi_{ij}$ ‘s to be multinomial probabilities by removing the constraint that $f_{1k}+\cdots +f_{mk}=1$, which allows us to analyze the genes j=1,…,m independently. This is a good approximation so long as $s_{i}$ is large and the $f_{jk}$’s are small (the same sort of approximation was used for GLM-PCA [8]). To be explicit about this approximation, we make the approximations $\pi_{ij}\approx \theta_{ij}$ (which are no longer guaranteed to be multinomial probabilities) and $f_{jk}\approx p_{jk}$ (which are no longer guaranteed to sum to one), resulting in the GoM DE model, which for convenience we restate here:

(9) $$x_{ij}\sim Poisson\left(s_{i}\theta_{ij}\right),\;\theta_{ij}=\sum_{k=1}^{K}\;\;l_{ik}p_{jk}.$$

#### “Null” model

The simplest Poisson model of the form (9) is one in which $\theta_{ij}$ is the same across all cells i; that is, $\theta_{ij}=p_{j0}$ for all i=1,…,n. We treat this a “null” model, which can be used to make certain comparisons, e.g., to estimate changes in expression in relative to the average expression of gene j across all cells. The maximum-likelihood estimate (MLE) of $p_{j0}$ under the null model is

(10) $${\overset{ˆ}{p}}_{j0}=\frac{\sum_{i=1}^{n}\;\;x_{ij}}{\sum_{i=1}^{n}\;\;s_{i}}.$$

#### Two-group model

The next simplest Poisson model of the form (9) is one in which the the cells are split into two groups, and $\theta_{ij}$ is determined by the cell’s membership to the two groups. Introducing binary latent variables $z_{ij}\in \{1,2\}$ to denote group membership, we define $\theta_{ij}=p_{j1}$ if $z_{i}=1$, otherwise $\theta_{ij}=p_{j2}$. Under this two-group model, the log-fold change comparing group 1 to group 2 is

(11) $$\mathrm{LFC}\left(j\right)=\log_{2}\frac{p_{j1}}{p_{j2}}.$$

To estimate the LFC, we could compute, for example, MLEs of $p_{j1}$ and $p_{j2}$, which are

(12) $${\overset{ˆ}{p}}_{j1}\;=\frac{\sum_{i=1}^{n}\;\;\delta_{1}\left(z_{ij}\right)x_{ij}}{\sum_{i=1}^{n}\;\;\delta_{1}\left(z_{ij}\right)s_{i}}\;{\overset{ˆ}{p}}_{j2}\;=\frac{\sum_{i=1}^{n}\;\;\delta_{2}\left(z_{ij}\right)x_{ij}}{\sum_{i=1}^{n}\;\;\delta_{2}\left(z_{ij}\right)s_{i}},$$

where $\delta_{y}(x)=1$ when x=y, otherwise $\delta_{y}(x)=0$.

### Estimation of log-fold change

With more than two groups, it is less obvious how to define the log-fold change. To allow for some flexibility, we consider three possible LFC statistics. One possibility is to compare with the null model:

(13) $${\mathrm{LFC}}_{k}^{\text{null}}\left(j\right):=\log_{2}\frac{p_{jk}}{p_{j0}}.$$

Alternatively, we can compare one topic with another; see (4). A third possibility is the “least extreme” LFC (6).

This *grade-of-membership DE* (“GoM DE”) analysis is inherently a joint analysis over the K unknowns $p_{j1},\ldots ,p_{jK}$. A joint analysis over all K topics is needed to account for possible interdependencies among the topics. The GoM DE analysis is therefore more challenging to think about and implement than a comparison between two groups.

#### Maximum-likelihood estimation

A special convenience of the Poisson model allowing for grades of membership (5) is that we can reuse Poisson NMF computations (as described below, and in more detail in [48]) to compute MLEs of the unknowns $p_{jk}$: if we consider all genes j=1,…,m simultaneously, we recover a Poisson NMF model, $x_{ij}\sim Poisson\left(\lambda_{ij}\right),\;\lambda_{ij}=\sum_{k=1}^{K}\;h_{ik}w_{jk}$, by setting $h_{ik}=s_{i}l_{ik},\;w_{jk}=p_{jk}$. Therefore, we can reuse the Poisson NMF algorithms to compute MLEs of the unknowns $p_{jk}$.

#### Maximum a posteriori estimation

To improve numerical stability in the parameter estimation, we compute *maximum a posteriori* (MAP) estimates of $p_{j1},\ldots ,p_{jK}$, in which each $p_{jk}$ is assigned a gamma prior, $p_{jk}\sim Gamma(\alpha ,\beta )$, with α=1+ε,β=1, and ε>0. Typically ε will be some small, positive number, e.g., ε=0.01. Here we use the parameterization of the gamma distribution from [117] in which α is the shape parameter and β is the inverse scale parameter; under this parameterization, the mean is α/β and the variance is $\alpha /\beta^{2}$. The maximum-likelihood computations can be reused for MAP estimation with this gamma prior by adding “pseudocounts” to the data; specifically, MAP estimation of $p_{j1},\ldots ,p_{jK}$ given counts $x_{1j},\ldots ,x_{nj}$ and topic proportions L and is equivalent to maximum-likelihood estimation of $p_{j1},\ldots ,p_{jK}$ given counts $x_{1j},\ldots ,x_{nj},\varepsilon ,\ldots ,\varepsilon$ and topic proportions matrix $\left[\begin{array}{c} L\\ I_{K} \end{array}\right]$, where $I_{K}$ is the K×K identity matrix. Unless otherwise stated, we added ε=0.1 pseudocounts to the data.

#### Quantifying uncertainty and stabilizing LFC estimates

We implemented a simple Markov chain Monte Carlo (MCMC) algorithm [118, 119] to quantify uncertainty in the LFC estimates. Although MCMC is typically computationally intensive, with careful implementation (e.g., use of sparse matrix operations and multithreaded computations) the MCMC algorithm is quite fast. Other benefits of using MCMC is that the algorithm can straightforwardly accommodate different choices of LFC statistics, and no normality assumptions are needed.

The basic idea behind the MCMC algorithm is simple: for a given gene j, simulate the posterior distribution of the LFC statistic by performing a “random walk” on $g_{j}=\left(g_{j1},\ldots ,g_{jK}\right)$, where $g_{jk}:=logp_{jk},\;k=1,\ldots ,K$. The random walk generates a sequence of states $g_{j}^{1},\ldots ,g_{j}^{\left(n_{s}\right)}$, in which $n_{s}$ denotes the prespecified length of the simulated Markov chain. After choosing an initial state $g_{j}^{\left(0\right)}$, each new state $g_{j}^{\left(s+1\right)}$ is generated from the current state $g_{j}^{\left(s\right)}$ by the following procedure: first, a topic k∈{1,…,K} is chosen uniformly at random; next, a proposed state $g_{j}^{*}$ is generated as $g_{jk}^{*}=g_{jk}+\delta ,\delta \sim N\left(0,\sigma^{2}\right)$, with $g_{jk^{\prime }}^{*}=g_{jk^{\prime }}$ for all $k^{\prime }\neq k$. Assuming an (improper) uniform prior for the unknowns, $Pr\left(p_{jk}\right)\propto 1$, the proposed state is accepted into the Markov chain with probability

(14) $$𝒜(g_{j}^{\left(s\right)},g_{j}^{*})=\left\{1,\frac{Pr(x_{j}|p_{j}^{*})}{Pr(x_{j}|p_{j}^{\left(s\right)})}\times \frac{p_{jk}^{*}}{p_{jk}^{\left(s\right)}}\right\},$$

in which $x_{j}$ is the j th column of the counts matrix $X,\;x_{j}=\left(x_{1j},\ldots ,x_{nj}\right)$, and $Pr\left(x_{j}|p_{j}\right)$ is the likelihood at $p_{j},Pr\left(x_{j}|p_{j}\right)=\prod_{i=1}^{n}\;Poisson\left(x_{i};s_{i}\theta_{i}\right)$. (Note that $x_{j}$ may include pseudocounts.) The standard deviation of the Gaussian proposal distribution, σ, is a tuning parameter. (Unless otherwise stated, we use =0.3) The additional $p_{jk}^{*}/p_{jk}^{\left(s\right)}$ term in the acceptance probability is needed to account for the fact that we are simulating the log-transformed parameters $g_{j}$, and not $p_{j}$ [120, p. 11]. When the proposal is not accepted, the new state is simply copied from the previous state, $g_{j}^{\left(s+1\right)}=g_{j}^{\left(s\right)}$.

Most of the computational effort in running the MCMC goes into computing the acceptance probabilities (14), so we have carefully optimized these computations. For example, we have taken advantage of the fact that the count vectors $x_{j}$ are typically very sparse. Additionally, these computations can be performed in parallel since the Markov chains are simulated independently for each gene j.

Once Monte Carlo samples $g^{\left(s\right)}$, for $s=1,\ldots ,n_{s}$, have been simulated by this random-walk MCMC, we compute posterior mean LFC estimates, and quantify uncertainty in the LFC estimates. For example, expressing the least extreme LFC for gene j and topic k as a function of the unknowns, ${LFC}_{k}^{\text{l.e.}}\left(p_{j}\right)$, the posterior mean least extreme LFCs are calculated as $E[{LFC}_{k}^{\text{l.e.}\;}\left(p_{j}\right)]\approx \sum_{s=1}^{n_{s}}\;{LFC}_{k}^{\text{l.e.}}(p_{j}^{\left(s\right)})$.

The final step in the GoM DE analysis is to perform adaptive shrinkage [82] to stabilize the posterior mean estimates. To implement this step, we use the ash function from the ashr R package [121]. We use the same settings as DESeq2 to replicate as closely as possible the performance of DESeq2 with adaptive shrinkage. DESeq2 calls ash with method = “shrink”, which sets the prior to be a mixture of uniforms without a point-mass at zero. (In some settings, other priors might be preferred, and ash allows for a variety of prior choices.)

The adaptive shrinkage method takes as input a collection of effect estimates ${\overset{ˆ}{\beta }}_{1},\ldots ,{\overset{ˆ}{\beta }}_{m}$ and associated standard errors ${\overset{ˆ}{s}}_{1},\ldots ,{\overset{ˆ}{s}}_{m}$. In this setting, it is not immediately obvious what are the standard errors, in part because the posterior distribution of the unknowns is not always symmetric about the mean or median. To provide a reasonable substitute summarizing uncertainty in the estimates, we compute Monte Carlo estimates of highest posterior density (HPD) intervals [122]. Specifically, let $\left[a_{jk},b_{jk}\right]$ denote the HPD interval for the LFC estimate of gene j in topic k, and let ${\overset{ˆ}{\beta }}_{jk}$ denote the posterior mean. We define the standard error as ${\overset{ˆ}{s}}_{jk}=b_{jk}-\mu_{jk}$ when ${\overset{ˆ}{\beta }}_{jk}<0$; otherwise, ${\overset{ˆ}{s}}_{jk}=\mu_{jk}-a_{jk}$. Defining the standard errors in this way prevents overshrinking of estimates that are uncertain but have little overlap with zero. We set the size of the HPD intervals to α=0.68 so that ${\overset{ˆ}{s}}_{jk}$ as we defined it will recover conventional standard error calculations when the posterior distribubtion is well approximated by the normal distribution. The revised posterior means and standard errors returned by the adaptive shrinkage method are then used by ashr to calculate test statistics including posterior z-scores (defined as the posterior mean divided by the posterior standard error [123]), local false sign rates (*lfsr*) and *s*-values, which are then used to report results of the GoM DE analysis.

A key question with MCMC how to choose *n_s_*; that is, how long a Markov chain to simulate. One heuristic way to assess whether *n_s_* is large enough is to perform two independent MCMC runs initialized with different pseudorandom number generator states (“seeds”), and check consistency of the posterior estimates from the two runs. (We checked consistency of the posterior estimates after stabilizing the estimates using adaptive shrinkage, as described above.) Using simulated data sets (see below), comparison of the two independent MCMC runs suggests that $n_{s}=10,000$ was sufficient to obtain reasonably accurate estimates of posterior means and posterior *z*-scores (Additional file 1: Figure S6). We also performed a similar assessment in the PBMC data; in the PBMC data, we found that good consistency was achieved with $n_{s}=100,000$ (Additional file 1: Figure S5). Therefore, unless otherwise stated we used $n_{s}=100,000$ in the GoM DE analyses.

The GoM DE analysis methods are implemented in the de_analysis function in the fastTopics package.

### Single-cell data sets

All data sets analyzed were stored as sparse n×m matrices X, where n is the number of cells and m is the number of genes or regions.

#### Preparation of scRNA-seq data

Since the topic model is a multinomial model of count data, no log-normalization or other transformation of the scRNA-seq molecule counts is needed. Further, we retain all genes other than those with no variation in the data set. This is done in part to demonstrate that our methods are robust to including genes with little variation. Also note that due to the use of sparse matrix techniques in our software implementations, including genes with low variation does not greatly increase computational effort.

#### Preparation of scATAC-seq data

As has been previously suggested [e.g., 19, 74–76]), we “binarized” the single-cell ATAC-seq data; that is, we assigned $x_{\text{ij}}=1$ (“accessible”) when least one fragment in cell *i* maps to peak or region *j*, otherwise $x_{\text{ij}}=0$ (“inaccessible”). There are at least a couple reasons for doing this. For small peaks (say, < 5 kb), read counts do not provide a reliable quantitative measure of accessibility in single cells. This is because the (random) first insertion restricts the space for subsequent insertions. Additionally, insertions could occur within the same site on the same allele or on each of the two alleles, complicating interpretation of the read counts.

Like the RNA molecule count data (see above), we retain all regions except those that show no variation.

#### PBMC data from Zheng *et al*, 2017

We combined reference transcriptome profiles generated from 10 bead-enriched subpopulations of PBMCs (Donor A) [29]. Specifically, we downloaded the “Gene/cell matrix (filtered)” tar.gz file from the 10x Genomics website for each of the following 10 FACS-purified data sets: CD14+ monocytes, CD19+ B cells, CD34+ cells, CD4+ helper T Cells, CD4+/CD25+ regulatory T Cells, CD4+/CD45RA+/CD25− naive T cells, CD4+/CD45RO+ memory T Cells, CD56+ natural killer cells, CD8+ cytotoxic T cells and CD8+/CD45RA+ naive cytotoxic T cells. After combining these 10 data sets, then filtering out unexpressed genes, the combined data set contained molecule counts for 94,655 cells and 21,952 genes; 97.1% of the molecule counts were zero. In Fig. 1, the 54,132 cells from these data sets were labeled as “T cells”: CD4+ helper T Cells, CD4+/CD25+ regulatory T Cells, CD4+/CD45RA+/CD25− naive T cells, CD4+/CD45RO+ memory T Cells and CD8+/CD45RA+ naive cytotoxic T cells.

#### Epithelial airway data from Montoro *et al*, 2018

We analyzed a mouse epithelial airway data set from [87]: gene expression profiles of trachea epithelial cells in C57BL/6 mice obtained using dropletbased 3’ scRNA-seq, processed using the GemCode Single Cell Platform. The data were downloaded from the Gene Expression Omnibus (GEO) website, accession GSE103354; specifically, we downloaded file GSE103354 Trachea droplet_UMIcounts.txt.gz (This file also contains the cluster assignments that we compare against our results; in [87], the samples were subdivided into 7 clusters using a community detection algorithm.) After removing genes that were not expressed in any of the cells, the data set contained molecule counts for 7,193 cells and 18,388 genes (90.7% of counts were zero).

#### Mouse Atlas data from Cusanovich *et al*, 2018

Cusanovich *et al* [98] profiled chromatin accessibility by single-cell combinatorial indexing ATAC-seq (sciATAC-seq [124, 125]) in nuclei from 13 distinct tissues of a 8-week-old male C57BL/6J mouse. Replicates for 4 of the 13 tissues were obtained by profiling chromatin accessibility in a second mouse. We downloaded the (sparse) binarized peak × cell matrix in RDS format, atac_matrix.binary.qc_filtered.rds, from the Mouse sci-ATAC-seq Atlas website, https://atlas.gs.washington.edu/mouse-atac/data. We also downloaded cell_metadata.txt, which included the cell types estimated using a clustering of the cells (see Table S1 in [98]). The full data set used in our analysis (13 tissues including 4 replicated tissues) consisted of the binary accessibility values for 81,173 cells and 436,206 peaks (1.2% overall rate of accessibility). (Note that all of the peaks had fragments mapping to at least 40 cells, so no extra step was taken to filter out peaks.) Separately, we analyzed the sci-ATAC-seq data from kidney only, in which peaks with fragments mapping to fewer than 20 kidney cells were removed, resulting in data set containing binary accessibility values for 6,431 cells and 270,864 peaks. Base-pair positions of the peaks are based on Mouse Genome Assmebly mm9 (NCBI and Mouse Genome Sequencing Consortium, Build 37, July 2007).

From the Mouse sci-ATAC-seq website, we also downloaded the file master_cicero_conns.rds containing the Cicero co-accessibility predictions [96], which we used to link chromatin accessibility peaks to genes. (For details on how these Cicero predictions were obtained from the sci-ATAC-seq data, see [98].) For the kidney data, we connected a peak given in the “Peak2” column of the Cicero co-accessibility data table to a gene given in the “peak1.tss.gene_id” column if the “cluster” column was 11, 18, 22 or 25 (these four clusters were the main kidney-related clusters identified in [98]). This extracted, for each gene, the distal and proximal sites connected to the gene associated with Peak1 (specifically, a gene in which the transcription start site overlaps with Peak1). Among the 22,194 genes associated with at least one peak, the median number of peaks connected to a gene was 19, and the largest number of peaks was 179 (for *Bahcc1* on chromosome 11). Among the 270,864 peaks included in the topic modeling analysis, 113,489 (42%) were connected to at least one gene, 95% of peaks were connected to 10 genes or fewer, and the largest number of connected genes was 60.

#### Human hematopoietic system data from Buenrostro *et al*, 2018

Buenrostro *et al* [105] used FACS to isolate 10 hematopoietic cell populations from human bone marrow and blood, then the cells were assayed using single-cell ATAC-seq. The processed single-cell ATAC-seq data were downloaded from the GEO website, accession GSE96772; specifically, file GSE96769_scATACseq_-counts.txt.gz containing the fragment counts, and file GSE96769_PeakFile_20160207.bed.gz containing peaks obtained from bulk ATAC-seq data [105]. Although there may be benefits to calling peaks using aggregated single-cell data instead [126], we used the original accessibility data based on the bulk ATAC-seq peaks so that our analysis was more directly comparable to the analysis of [105].

Following [105, 126], we extracted the 2,034 samples passing quality control filters, then we “binarized” the counts; that is, we set $x_{\text{ij}}=1$ when at least one fragment in cell *i* mapped to region *j*; otherwise, $x_{\text{ij}}=0$. The list of 2,034 cells considered “high quality” was obtained from file metadata.tsv included in the online benchmarking repository [126]. After removing peaks with fragments mapping to fewer than 20 cells, the final data set used in our analysis consisted of binary accessibility values for 2,034 cells and 126,719 peaks (4.6% overall rate of accessibility). Base-pair positions of the peaks were based on human genome assembly 19 (Genome Reference Consortium Human Build 37, February 2009).

In [105], a large, patient-specific batch effect was identified in the accessibility profiles for the HSC cells, and therefore steps were taken in [105] to normalize the accessibility data before performing a principal component analysis. We instead fit the topic model to the raw binary accessibility values, in part to find out how well the topic model can cope with the complication of a batch effect. In agreement with [105], this batch effect is at least partly captured by the topics, although in our analysis the batch effect appeared also in MPP cells and, to a lesser extent, in CMP cells (Additional file 1: Figure S15).

### Fitting the topic models

In brief, we took the following steps to fit a topic model. These steps are all implemented in the fastTopics R package.

First, we fit a Poisson NMF model [37, 127],

(15) $$x_{ij}\;\sim Poisson\left(\lambda_{ij}\right)\;\Lambda ={HW}^{T},$$

where $\Lambda \in R^{n\times m}$ is a matrix of the same dimension as X, with entries $\lambda_{ij}\geq 0$ giving the Poisson rates for the counts $x_{ij}$. The parameters of the Poisson NMF model are stored as two matrices, $H\in R^{n\times K},\;W\in R^{m\times K}$, with non-negative entries $h_{ik},\;w_{jk}\geq 0$. fastTopics has efficient implementations of algorithms for computing maximum-likelihood estimates (MLEs) of W,H.

Second, we recover MLEs of F,L from MLEs of W,H by a simple reparameterization [48].

In an empirical comparison of Poisson NMF algorithms with count data sets, including scRNA-seq data sets, [48], we found that a simple co-ordinate descent (CD) algorithm [128, 129], when accelerated with the extrapolation method of Ang and Gillis [130], almost always produced the best Poisson NMF (and topic model) fits, and in the least amount of time. To confirm this, we compared topic model fits obtained by running the same four algorithms that were compared in [48]—EM and CD, with and without extrapolation—on the PBMC data set, and assessed the quality of the fits. We evaluated the model fits in two ways: using the likelihood, and using the residuals of the Karush-Kuhn-Tucker (KKT) first-order conditions (the residuals of the KKT system should vanish as the algorithm approaches maximum-likelihood estimates of W,H). Following [48], to reduce the possibility that multiple optimizations converge to different local maxima of the likelihood, which could complicate these comparisons, we first ran 1,000 EM updates, then we examined the performance of the algorithms after this initialization phase (Additional file 1: Figures S2, S3). Consistent with [48], the extrapolated CD updates always produced the best fit, or at the very least a fit that was no worse than the other algorithms, and almost always converged on a solution more quickly than the other algorithms. Therefore, subsequently we used the extrapolated CD updates to fit the topic models.

In more detail, the pipeline for fitting topic models consisted of the following steps: (1) Initialize W using Topic-SCORE [131]; (2) perform 10 CD updates of H, with W fixed; (3) perform 1,000 EM updates (without extrapolation) to get close to a solution (“prefitting phase”); (4) run an additional 1,000 extrapolated CD updates to improve the fit (“refinement phase”); and (5) recover F,L from W,H. The prefitting phase was implemented by calling fit_poisson_nmf from fastTopics with these settings: numiter = 1000, method = “em”, control = list(numiter = 4). The refinement phase was implemented with a second call to fit_poisson_nmf, with numiter = 1000, method = “scd”, control = list(numiter = 4,extrapolate = TRUE), in which the model fit was initialized using the fit obtained from the prefitting phase. The topic model fit was recovered by calling poisson2multinom in fastTopics. Note that only the estimates of L were needed for the GoM DE analysis.

For each data set, we fit topic models with different choices of *K* and compared the fits for each *K* by comparing their likelihoods (Additional file 1: Figure S1).

### Visualizing the topic proportions

The topic proportions matrix L can be viewed as an embedding of the cells i=1,…,n in a (K-1) dimensional space [50]. (It is K-1 dimensions because of the constraint that the topic proportions for each cell must add up to 1.) A simple way to visualize this embedding in 2-d is to apply a nonlinear dimensionality reduction technique such as t-SNE [11, 132] or UMAP [12] to L ([49] used t-SNE). We have also found that plotting principal components (PCs) of the topic proportions can be an effective way to explore the structure inferred by the topic model (see Additional file 1: Figures S4, S8 for examples). However, we view these visualization techniques as primarily for exploratory ends, and a more powerful approach is to visualize all *K* − 1 dimensions simultaneously using a Structure plot [70, 71]. Here we describe some improvements to the Structure plot for better visualization of single-cell data sets. The improvements described here are implemented by the structure_plot function in fastTopics.

When cells were labeled, we compared topics against labels by grouping the cells by label in the Structure plot (e.g., Fig. 1B). To effectively visualize the topic proportions within each group, we applied *t*-SNE to L to arrange the cells on a line (specifically, we use R package Rtsne [133]). In fastTopics we also implemented the option to arrange the cells in each group using UMAP or PCA, which are typically faster than *t*-SNE, but in our experience we found that UMAP and PCA produced “noisier” visualizations.

Arranging the cells by 1-d *t*-SNE worked best for smaller groups of cells with less complex structure. For large groups of cells, or for unlabeled single-cell data sets, we randomly subsampled the cells to reduce *t*-SNE runtime (when cells number in the thousands, it is nearly impossible to distinguish individual cells in the Structure plot anyhow). Even with this subsampling, the Structure plot sometimes did not show fine-scale substructures or rare cell types. Therefore, in the more complex cases, we first subdivided the cells into smaller groups based on the topic proportions, then ran *t*-SNE on these smaller groups. The smaller groups were either identified visually from PCs of L, or in a more automated way by running *k*-means on PCs of L (as suggested in [134]). For example, this was the approach taken to generate the Structure plots in Fig. 1A (split into groups a–g) and Fig. 6A (“abundant”, “rare”).

### Gene enrichment analysis based on differential accessibility of peaks connected to genes

Here we describe a simple approach to obtain gene-level statistics from the results of a differential accessibility analysis. This approach was applied in the topic modeling analysis of the Mouse Atlas kidney cells to help interpret the topics.

Cusanovich *et al* [98] used the Cicero co-accessibility predictions and the binarized single-cell ATAC-seq data to compute a “gene activity score” *R_ki_* for each gene *k* and cell *i*. Here we have a related but different goal: we would like to use the results of the differential accessibility analysis, which generates differential accessibility estimates and related statistics for each peak and each topic, to rank genes according to their importance to a given topic. A difficulty, however, with ranking the genes is that the Cicero co-accessibility predictions are uncertain, and they are only partially informative about which peaks are relevant to a gene. In aggregate, however, the expectation is that the “most interesting” genes will be genes that are (a) predicted by Cicero to be connected many peaks that are differentially accessible and (b) the differences in accessibility are mainly in the same direction. This suggests an enrichment analysis in which, for each gene, we test for enrichment of differential acccessibility among the peaks connected to that gene. Here we describe a simple enrichment analysis in which we derive and compute statistical quantities for (a) and (b). For (a), we computed a Bayes factor [135] measuring the support for the hypothesis that at least one of the peaks is differentially accessible (the LFC is not zero) against the null hypothesis that none of the peaks are differentially accessible. For (b), we computed the *average LFC* among all differentially accessible peaks (that is, peaks with nonzero LFC according to some significance criterion).

We implemented this gene enrichment analysis by running adaptive shrinkage [82] separately for each gene and topic. This had the benefit of adapting the shrinkage separately to each gene in each topic. In particular, in comparison to the usual adaptive shrinkage step for a GoM DE analysis (see above), it avoided overshrinking differences for genes exhibiting strong patterns of differential accessibility. We took the following steps to implement this adaptive shrinkage analysis. First, we ran function ash from the ashr package [121] once on the posterior mean l.e. LFC estimates and ${\overset{ˆ}{\beta }}_{jk}$ their standard errors ${\overset{ˆ}{s}}_{jk}$ for all topics k and all peaks j, with settings mixcompdist = “normal”, method = “shrink”. (Note that the usual adaptive shrinkage step for the GoM DE analysis was skipped.) This was done only to determine the variances in the mixture prior and to get a “default” model fit to be used in the subsequent adaptive shrinkage analyses.

Next, we ran, separately for gene and each topic k, ash using the LFC estimates ${\overset{ˆ}{\beta }}_{jk}$ and standard errors ${\overset{ˆ}{s}}_{jk}$ from the peaks j connected to that gene. We set the variances in the mixture prior to the variances determined from all the LFC estimates, and used ash settings mixcompdist = “normal” and pointmass = FALSE. One issue with running adaptive shrinkage using only the LFC estimates for the peaks connected to a gene is that some genes have very few Cicero connections, leading to potentially unstable fits and unreliable posterior estimates. We addressed this issue by encouraging the fits toward the “default model” that was fitted to all genes and all topics; specifically, we set the Dirichlet prior on the mixture proportions to be Dirichlet $\left(\alpha_{1},\ldots ,\alpha_{K}\right)$ with prior sample sizes $\alpha_{k}=1.01+n_{0}{\overset{ˆ}{\pi }}_{k}^{\text{default}}$, where here K denotes the number of components of the prior mixture (not the number of topics), ${\overset{ˆ}{\pi }}_{k}^{\text{default}}$ denotes the *k*th mixture proportion in the adaptive shrinkage prior for the fitted “default” model, and $n_{0}=20$. This stabilized the model fits for genes with few Cicero connections, while still giving some freedom for genes with many connections to adapt the model to the data.

Finally, we used the logLR output from ash as a measure of support for enrichment (this is the Bayes factor on the log-scale), and we computed the mean l.e. LFC as the average of the posterior mean estimates of the l.e. LFCs taken over all peaks j connected to the gene and with *lfsr* < 0.05.

### Motif enrichment analysis for differentially accessible (DA) regions

We used HOMER [106] to identify transcription factor (TF) motifs enriched for differentially accessible regions, separately for each topic estimated in the single-cell ATAC-seq data. For each topic k=1,…,K, we applied the HOMER Motif Analysis tool findMotifsGenome.pl to estimate motif enrichment in differentially accessible regions; specifically, we took “differentially accessible regions” to be those with *p*-value less than 0.05 in the GoM DA analysis. These differentially accessible regions were stored in a BED file positions.bed. The exact call from the commandline shell was findMotifsGenome.pl positions.bed hg19 homer -len 8,10,12 -size 200 -mis 2 -S 25 -p 4 -h.

Note that the adaptive shrinkage step was skipped in the GoM DA analysis, so these are the *p*-values for the unmoderated least extreme LFC estimates. The reason for skipping the adaptive shrinkage step is that the shrinkage is performed uniformly for the LFC estimates for all regions, and since the vast majority of regions have LFC estimates are that are indistinguishable from zero, the result is that very few differentially accessible regions remain after performing adaptive shrinkage. (One would ideally want to incorporate the enrichment about motif enrichment into the adaptive shrinkage step.)

### Gene sets

Human and mouse gene sets for the gene set enrichment analyses (GSEA) were compiled from the following gene set databases: NCBI BioSystems [136]; Pathway Commons [137, 138]; and MSigDB [139–141], which includes Gene Ontology (GO) gene sets [95, 142]. Specifically, we downloaded bsid2info.gz and biosystems_gene.gz from the NCBI FTP site (https://ftp.ncbi.nih.gov/gene) on March 22, 2020; PathwayCommons12.All.hgnc.gmt.gz from the Pathway Commons website (https://www.pathwaycommons.org) on March 20, 2020; and msigdb_v7.2.xml.gz from the MSigDB website (https://www.gsea-msigdb.org) on October 15, 2020. For the gene set enrichment analyses we also downloaded human and mouse gene information (“gene info”) files Homo_sapiens.gene_info.gz and Mus_musculus.gene_info.gz from the NCBI FTP site on October 15, 2020. Put together, we obtained 37,856 human gene sets and 33,380 mouse gene sets, although in practice we filtered out many gene sets based on certain criteria before running the GSEA (exact numbers of gene sets tested for enrichment in each gene set enrichment analysis are given below). To facilitate integration of these gene sets into our analyses, we have compiled these gene sets into an R package [143].

### Gene set enrichment analysis

We take a simple multiple linear regression approach to the gene set enrichment analysis (GSEA), in which we model the (least extreme) LFC estimate for gene i in a given topic, here denoted by $y_{i}$, as $y_{i}=\mu_{i}+$ $\sum_{j=1}^{n}\;x_{ij}b_{j}+e_{i},\;e_{i}\sim N\left(0,\sigma^{2}\right)$, in which $x_{ij}\in \{0,1\}$ indicates gene set membership; $x_{ij}=1$ if gene i belongs to gene set j, otherwise $x_{ij}=0$. (We represent the gene-set membership as a sparse matrix since most $x_{ij}$ ‘s are zero.) Here, n denotes the number of candidate gene sets, and $\sigma^{2}$ is the residual variance, which will be estimated. The idea behind this simple approach is that the most relevant gene sets are those that best explain the log-fold changes $y_{i}$, and therefore in the multiple regression we seek to identify these gene sets by finding coefficients $b_{j}$ that are nonzero with high probability. See [144, 145] for similar ideas using logistic regression. Additionally, since many genes are typically differentially expressed in a given topic—for example, a conservative estimate is that 1,995 genes are differentially expressed in the “B cells” topic (topic 1 in Fig. 2), with *lfsr* < 0.01—modeling LFC helps distinguish among DE genes that show only a slight increase in expression versus those that are highly overexpressed [146, 147]. Of course, this simple multiple linear approach ignores uncertainty in the LFC estimates $y_{i}$, which is accounted for in most gene set enrichment analyses (for example, for genes expressed at low levels, the LFC estimates will typically have high variance). We address this issue by shrinking the LFC estimates *prior to running the GSEA*; that is, we take $y_{i}$ to be the the posterior mean LFC estimate after applying adaptive shrinkage, as described above (see “Quantifying uncertainty and stabilizing LFC estimates”). This has the effect that genes that we are more uncertain about will have an LFC estimate $y_{i}$ that is zero or near zero.

We implemented the multiple linear regression approach using SuSiE (susieR version 0.12.10) [148]. A benefit to using SuSiE is that it automatically organizes similar or redundant gene sets into “credible sets” (CSs), making it easier to quickly recognize complementary gene sets; see [149–154] for related ideas.

In detail, the GSEA was performed as follows. We performed a separate GSEA for each topic, k=1,…,K. Specifically, for each topic k, we ran the susieR function susie with the following options: L = 10, intercept = TRUE, standardize = FALSE, estimate residual variance = TRUE, refine = FALSE, compute univariate zscore = FALSE and min abs corr = 0. We set L = 10 so that SuSiE returned at most 10 credible sets—that is, at most 10 enriched gene sets). For a given topic *k*, we reported a gene set as being enriched if it was included in at least one CS. We organized the enriched gene sets by credible set (specifically, 95% credible sets). We also recorded the Bayes factor for each CS, which gives a measure of the level of support for that CS. For each gene set included in a CS, we reported the posterior inclusion probability (PIP), and the posterior mean estimate of the regression coefficient $b_{j}$. In the results, we refer to $b_{j}$ as the “enrichment coefficient” for gene set j since it is an estimate of the expected increase in the LFC for genes that belong to gene set j relative to genes that do not belong to the gene set.

Often, a CS contained only one gene set, in which case the PIP for that gene set was close to 1. In several other cases, the CS contained multiple similar gene sets; in these cases, the smaller PIPs indicate that it is difficult to choose among the gene sets because they are similar to each other. (Note that the sum of the PIPs in a 95% CS should always be above 0.95 and less than 1.) Occasionally, SuSiE returned a CS with a small Bayes factor containing a large number of gene sets. We excluded such CSs from the results.

When repeated these gene set enrichment analyses with two collections of gene sets: (1) all gene sets other MSigDB collections C1, C3, C4 and C6, and “archived” MSigDB gene sets; and (2) only gene sets from curated pathway databases, specifically Pathway Commons, NCBI BioSystems, “canonical pathways” (CP) in the MSigDB C2 collection, and Gene Ontology (GO) gene sets in the MSigDB C5 collection. We removed gene sets with fewer than 10 genes and with more than 400 genes. Table 1 gives the exact number of gene sets included in each GSEA.

### Simulations

For evaluating the DE analysis methods, we generated matrices of UMI counts $X\in R^{n\times m}$ for n=200 cells and m=10,000 genes. We simulated the UMI counts $x_{ij}$ from a Poisson NMF model (15) in which W and H were chosen to emulate UMI counts from single-cell RNA sequencing experiments.

The matrices W and H were generated as follows. First, for each cell i, we generated topic proportions $l_{i1},\ldots ,l_{iK}$ then set $h_{ik}=s_{i}l_{ik}$, for k=1,…,K, where $s_{i}$ is the total UMI count. To simulate the wide range of total UMI counts often seen in scRNA-seq data sets, total UMI counts $s_{i}$ were normally distributed on the log-scale, $s_{i}=10^{u_{i}},\;u_{i}\sim N(0,1/5)$ where N(μ,σ) denotes the univariate normal distribution with mean μ and standard deviation σ.

Topic proportions $l_{ik}$ for each cell i were generated so as to obtain a wide range of mixed memberships, according to the following procedure: the number of nonzero proportions was set to $K^{\prime }\in \{1,\ldots ,K\}$ with probability $2^{-K^{\prime }}$; the $K^{\prime }$ selected topics $t_{1},\ldots ,t_{K^{\prime }}\subseteq \{1,\ldots ,K\}$ were drawn uniformly at random (without replacement) from 1,…,K; then the topic proportions for the selected topics were set to 1 when $K^{\prime }=1$, or, when $K^{\prime }>1$, they were drawn from the Dirichlet distribution with shape parameters $\alpha_{t_{1}},\ldots ,\alpha_{t_{K^{\prime }}}$.

Expression rates $w_{jk}$ were generated so as to emulate the wide distribution of gene expression levels observed in single-cell data sets, and to allow for differences in expression rates among topics. The procedure for generating the expression rates for each gene j was as follows: with probability 1/2, the expression rates were the same across all topics, and were generated as $f_{j1}=\cdots =f_{jK}=2^{v_{j}},\;v_{j}\sim N(-4,2)$. Otherwise, with probability 1/2, the expression rates were the same in all topics except for one topic, and the differing topic, $k^{\prime }$, was chosen uniformly at random from 1,…,K, then the expression rate for topic $k^{\prime }$ was set to $f_{jk^{\prime }}=2^{v_{j}+e_{j}},\;e_{j}\sim N(0,1)$.

Using this simulation procedure, we generated three sets of data sets. The simulation settings were altered slightly for each set. In the first set, data sets were simulated with K=2,α=(1/100,1/100) so that most topic proportions were equal or very close to 0 or 1. In the second set, we used K=2 and α=(1,1) to allow for a range of mixed memberships to topics. And in the third set, we generated data sets with K=6 topics and α=(1,…,1). As a result, the expression rates were roughly normally distributed on the logscale, and the expression differences were also normally distributed on the log-scale. About half of genes had an expression difference among the topics.

For each of the data sets simulated with K=2,α=(1/100,1/100) so that the cells in these data sets could essentially be subdivided into two groups, we ran MAST [85, 155] and DESeq2 [80, 84] to test for genes that were differentially expressed between the two groups. MAST (R package version 1.20.0) was called via the FindMarkers interface in Seurat [25] (Seurat 4.0.3, SeuratObject 4.0.2) with the following settings: ident.1 = “2”, ident.2 = NULL, test.use = “MAST”, logfc.threshold = 0, min.pct = 0. DESeq was called from the DESeq2 R package (version 1.34.0) using settings recommended in the package vignette: test = “LRT”, reduced = ~1, useT = TRUE, minmu = 1e-6, minReplicatesForReplace = Inf. Size factors were calculated using the calculateSumFactors method from scran version 1.22.1 [23]. The LFC estimates returned by DESeq were subsequently revised using adaptive shrinkage [82] by calling lfcShrink in DESeq2 with type = “ashr”, svalue = TRUE. (As in the GoM DE analysis, the DESeq2 posterior *z*-scores were defined as the posterior means divided by the posterior standard errors returned by the adaptive shrinkage.)

To perform the GoM DE analysis in each of the simulations, we first fit a Poisson NMF model to the simulated counts X using fit_poisson_nmf from the fastTopics R package [48, 156] (version 0.6–97). The loadings matrix H was fixed to the matrix used to simulate the data, and W was estimated by running 40 co-ordinate ascent updates on W alone (update.loadings = NULL, method = “scd”, numiter = 40). The equivalent topic model fit was then recovered. Three GoM DE analyses were performed using de_analysis from the fastTopics R package, with the topic model fit provided as input: one analysis without adaptive shrinkage (shrink.method = “none”), and two analyses with adaptive shrinkage (shrink.method = “ash”, ashr version 2.2–51 [121]) in which the MCMC was initialized with different pseudorandom number generator states. In all three runs, posterior calculations were performed with $n_{s}=10,000$, ε=0.01. Comparison of the two MCMC runs (with adaptive shrinkage) suggests that $n_{s}=10,000$ was sufficient to obtain reasonably accurate posterior estimates in these simulations (Additional file 1: Figure S6).

### Computing environment

Most computations on real data sets were run in R 3.5.1 [157], linked to the OpenBLAS 0.2.19 optimized numerical libraries, on Linux machines (Scientific Linux 7.4) with Intel Xeon E5–2680v4 (“Broadwell”) processors. For performing the Poisson NMF optimization, which included some multithreaded computations, as many as 8 CPUs and 16 GB of memory were used. The DESeq2 analysis of the PBMC data was performed in R 4.1.0, using 4 CPUs and 264 GB of memory. The evaluation of the DE analysis methods in simulated data sets was performed in R 4.1.0, using as many and 8 CPUs as 24 GB of memory.

## Supplementary Material

Supplement 1 Additional file 1: Supplementary Figures
Contains Supplementary Figures S1–S16.

Supplement 2 Additional file 2: Interactive volcano plots for PBMC data Interactive volcano plots for browsing the results from the GoM DE analyses of the PBMC data. Detailed l.e. LFC statistics are displayed on mouseover: lower and upper limit of HPD interval; posterior mean estimate; posterior *z*-score; and *lfsr*. The maximum-likelihood estimate of the expression rate, *p*_*j*0_, in the “null” expression model (10) is also given. Note that the lower and upper HPD intervals were not updated in the adaptive shrinkage step and therefore should be ignored.

Supplement 3 Additional file 3: Interactive volcano plots for epithelial airway data Interactive volcano plots for browsing the results from the GoM DE analyses of the mouse epithelial airway data. Detailed l.e. LFC statistics are displayed on mouseover: lower and upper limit of HPD interval; posterior mean estimate; posterior *z*-score; and *lfsr*. The maximum-likelihood estimate of the expression rate, *p*_*j*0_, in the “null” expression model (10) is also given. Note that the lower and upper HPD intervals were not updated in the adaptive shrinkage step and therefore should be ignored.

Supplement 4 Additional file 4: Tables S1, S2
Tables giving detailed statistics about DE genes identified in GoM DE analyses of PBMC (Table S1) and epithelial airway data (Table S2), with *lfsr* < 0.01. Columns in each table from left to right are: topic; ensembl id of gene (Table S1 only); HGNC gene symbol; posterior mean estimate of l.e. LFC; posterior *z*-score; and *lfsr*.

Supplement 5 Additional file 5: Tables S3–S6
Tables summarizing results of gene set enrichment analyses for PBMC (Tables S3, S4) and mouse epithelial airway data (Tables S5, S6). Columns in each table from left to right are: topic; credible set; log-Bayes factor (lbf); posterior inclusion probability (PIP); SuSiE posterior mean estimate of the enrichment coefficient (coef); gene set name; gene set id; database; accession, when applicable; sub-category code, when applicable; organism; brief description of the gene set; and a list of the 10 top genes, defined as the members of the gene set with the largest l.e. LFC (by magnitude).

Supplement 6 Additional file 6: Table S7
Table S7 giving detailed statistics about genes identified in the gene enrichment analysis for the Mouse Atlas kidney cells. All genes with log-Bayes factor > 17 are included in this table. (Here, we use the natural logarithm.) Assuming (quite conservatively) that 1 out of the 22,142 tested genes is enriched, this Bayes factor corresponds to a posterior odds (PO) of PO = *e*^17^/22,142 ≈ 1,000, or a posterior probability of about 0.999. Columns from left to right are: topic; gene symbol; Ensembl gene id; log-Bayes factor; average least extreme LFC among all peaks connected to the gene, with *lfsr* < 0.05.

Supplement 7 Additional file 7: Table S8
Table S8 giving results of the HOMER motif enrichment analysis for the Buenrostro *et al* human hematopoietic system data. Columns from left to right are: motif name; consensus sequence; and (base-10 log) enrichment *p*-value for each topic.

## Figures and Tables

**Table 1:** Number of gene sets included in each GSEA.

data set	all gene sets	curated only

PBMC	23,193	12,225
mouse epithelial airway	20,917	9,946
—rare epithelial cell types only	20,288	9,450
