# Supplementary material for: GoM DE: interpreting structure in sequence count data with differential expression analysis allowing for grades of membership
Source: bioRxiv. 2023 Sep 14:2023.03.03.531029. Originally published 2023 Mar 6. Preprint. [Version 3] doi: 10.1101/2023.03.03.531029 (PMC10028846; doi:10.1101/2023.03.03.531029)
Supplement: Supplement 4 — Additional file 4: Interactive volcano plots for PBMC data Interactive volcano plots for browsing the results from the GoM DE analyses of the PBMC data. Detailed l.e. LFC statistics are displayed on mouseover: lower and upper limit of HPD interval; posterior mean estimate; posterior z-score; and Ifsr. The maximum-likelihood estimate of the expression rate, pj0, in the “null” expression model (12) is also given. Note that the lower and upper HPD intervals were not updated in the adaptive shrinkage step and therefore should be ignored. [file media-4.zip › volcano_plots_pbmc/de_analysis_purified_pbmc_k1.html]

topic k1
