# Supplementary material for: Interpreting structure in sequence count data with differential expression analysis allowing for grades of membership
Source: bioRxiv. 2023 Jun 23:2023.03.03.531029. Originally published 2023 Mar 6. Preprint. [Version 2] doi: 10.1101/2023.03.03.531029 (PMC10028846; doi:10.1101/2023.03.03.531029)
Supplement: Supplementary file 1 [file media-1.pdf]

Supplementary figures for "Interpreting structure in sequence count data with differential expression analysis allowing for grades of membership"

Peter Carbonetto, Kaixuan Luo, Abhishek Sarkar, Anthony Hung, Sebastian Pott and Matthew Stephens

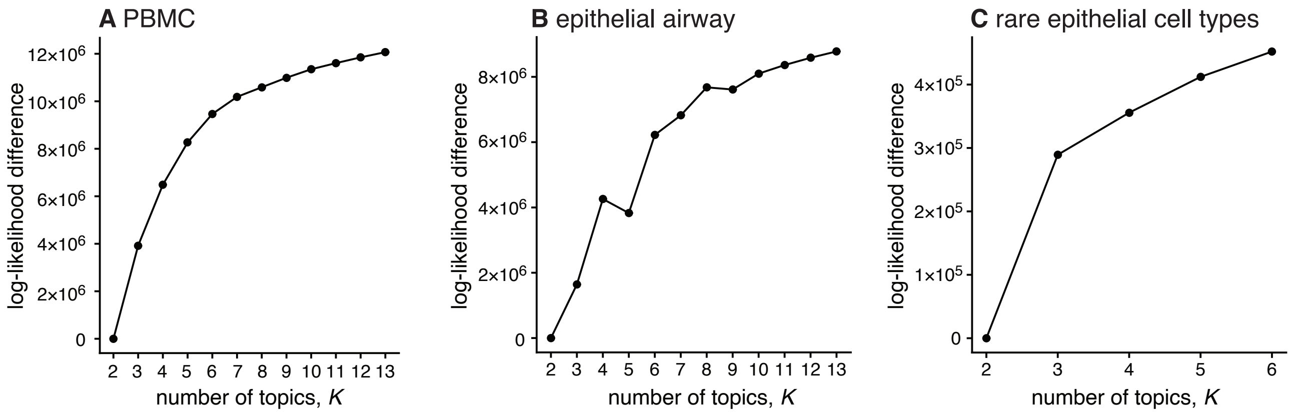

Fig. S1. Likelihood vs. K in (A) PBMC, (B) epithelial airway data, and (C) the rare cell type subset of epithelial airway cells. These plots shows topic model likelihoods for different choices of the number of topics, K. Log-likelihoods are shown relative to the log-likelihood at $K = 2$. Always the best likelihood is shown from the four optimization algorithms compared (EM and CD, with and without extrapolation). Note that these log-likelihood differences can either be interpreted as log-likelihood differences between two Poisson NMF model fits or between two topic model fits since the differences will be identical between two MLEs.

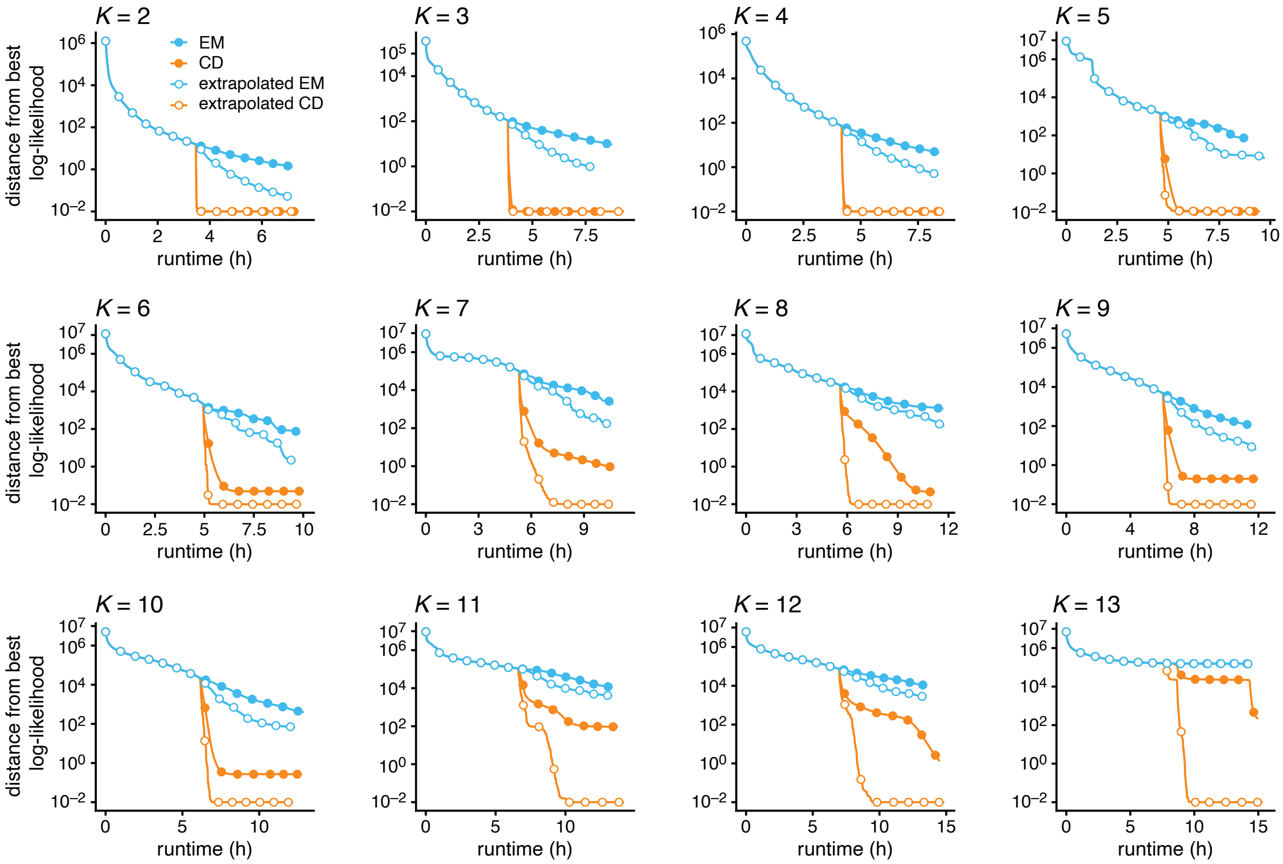

Fig. S2. Improvement in model fit over time for the Poisson NMF optimization algorithms applied to the PBMC data, with $K = 2, \ldots, 13$. Poisson NMF log-likelihoods are shown relative to the best log-likelihood recovered among the four algorithms compared (EM and CD, with and without extrapolation). All runs begin with a "pre-fitting" stage in which 1,000 EM updates are performed (with no extrapolation), followed by a "refinement" stage in which 1,000 updates are performed. Log-likelihood differences less than 0.01 are shown as 0.01. Circles are drawn at intervals of 150 iterations. Note on interpreting these plots: Poisson NMF log-likelihood differences shown in these plots are nearly identical to (multinomial) topic model log-likelihoood differences. This is because the Poisson NMF likelihoods are equal to the topic model likelihoods up to a proportionality constant so long as the size factors s_i are constant, and in practice the size factors do not change much because the optimization algorithms quickly converge to MLEs of the size factors.

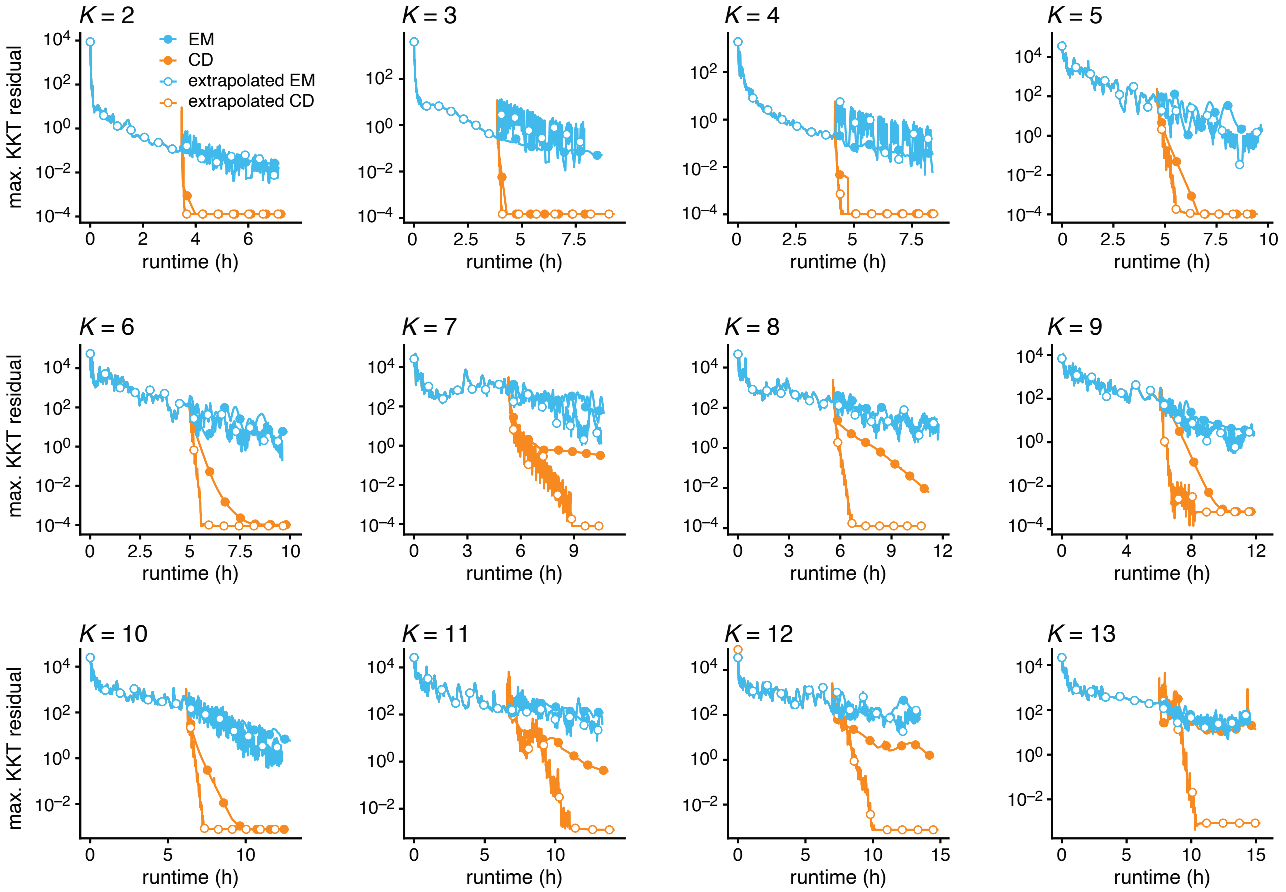

Fig. S3. Evolution of the KKT residuals over time for the Poisson NMF optimization algorithms applied to the PBMC data, with $K = 2, \ldots, 13$. All runs begin with a “pre-fitting” stage in which 1,000 EM updates are performed (with no extrapolation), followed by a “refinement” stage in which 1,000 updates are performed. Circles are drawn at intervals of 150 iterations. The KKT residuals should vanish near a local maximum of the likelihood, so looking at the largest KKT residual can be used to assess how well the algorithm recovers an MLE for the Poisson NMF or (multinomial) topic model. Note that, unlike the likelihood, the KKT residuals are not expected to decrease monotonically over time.

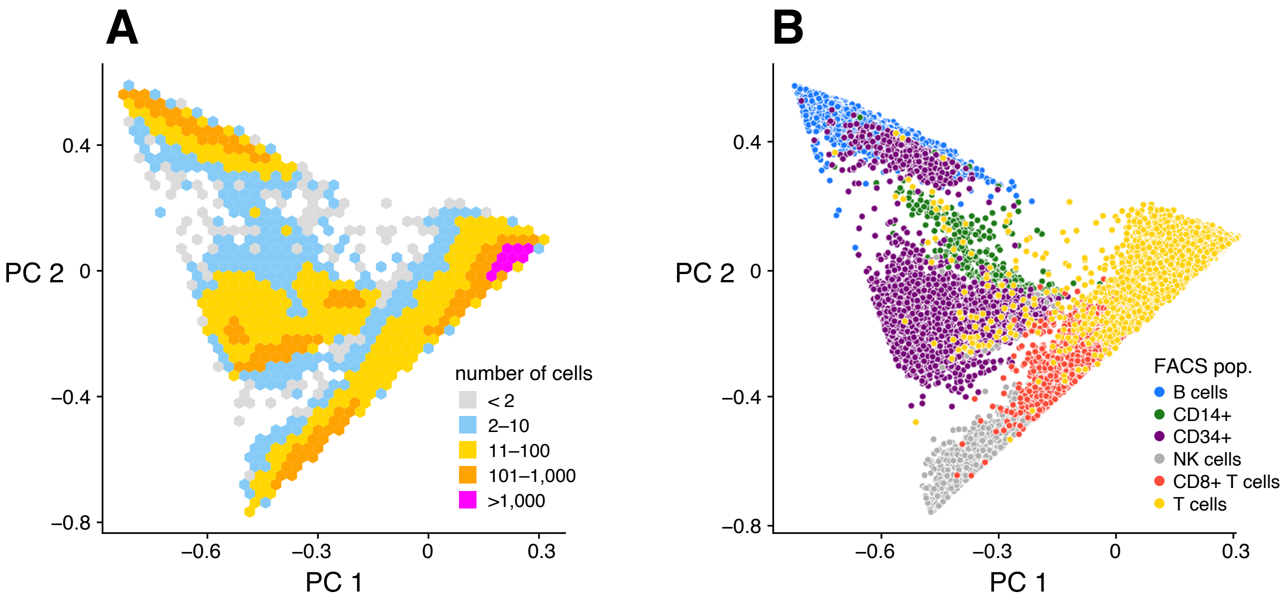

Fig. S4. Cells plotted by the first two PCs of the topic proportions matrix **L**. Panel A shows the density of the cells in this projection. In Panel B, cells are colored by FACS-purified subpopulation. The "T cells" label combines all purified T cell subpopulations other than CD8+ cytotoxic T cells.

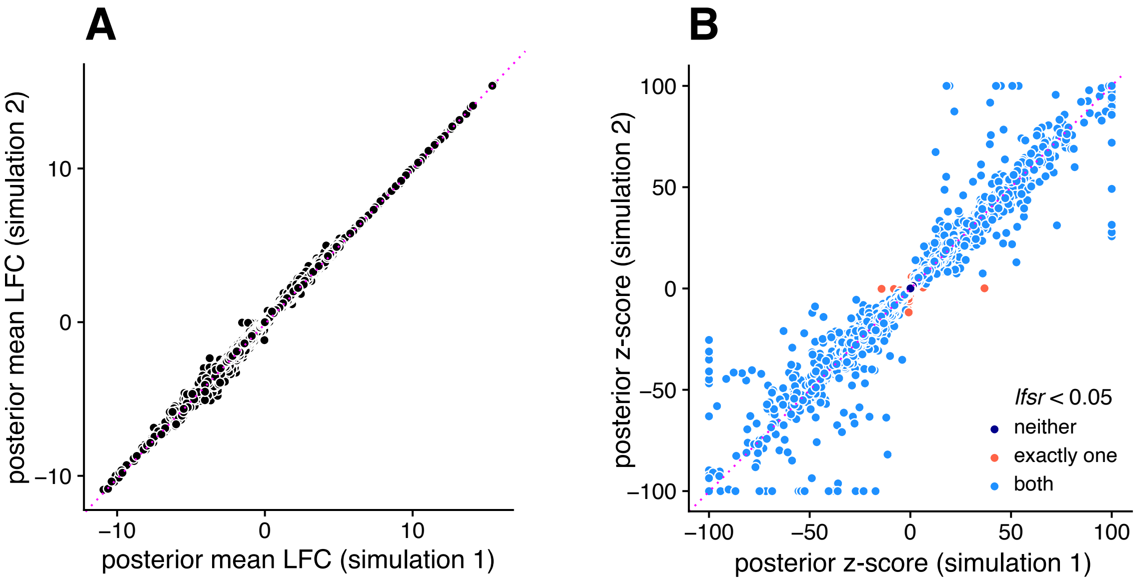

Fig. S5. Accuracy assessment of MCMC computations in PBMC data. To assess accuracy of the MCMC computations for the DE analysis, we performed two MCMC simulations with different random initializations (100,000 states were simulated in both MCMC runs). Panel A compares the posterior mean LFC estimates obtained from each run after performing adaptive shrinkage. Panel B compares the estimated posterior *z*-scores (again after performing adaptive shrinkage). Although the posterior *z*-scores are estimated less consistently than the posterior means the posterior *z*-scores are "consistent enough"; that is, it is rare for an LFC to have an *lfsr* less than 0.05 in one MCMC simulation and not in the other (the red points depict the LFCs with *lfsr* estimated inconsistently between the two runs). For better visualization of the posterior *z*-scores, posterior *z*-scores larger than 100 (or smaller than -100) are shown as 100 (or -100).

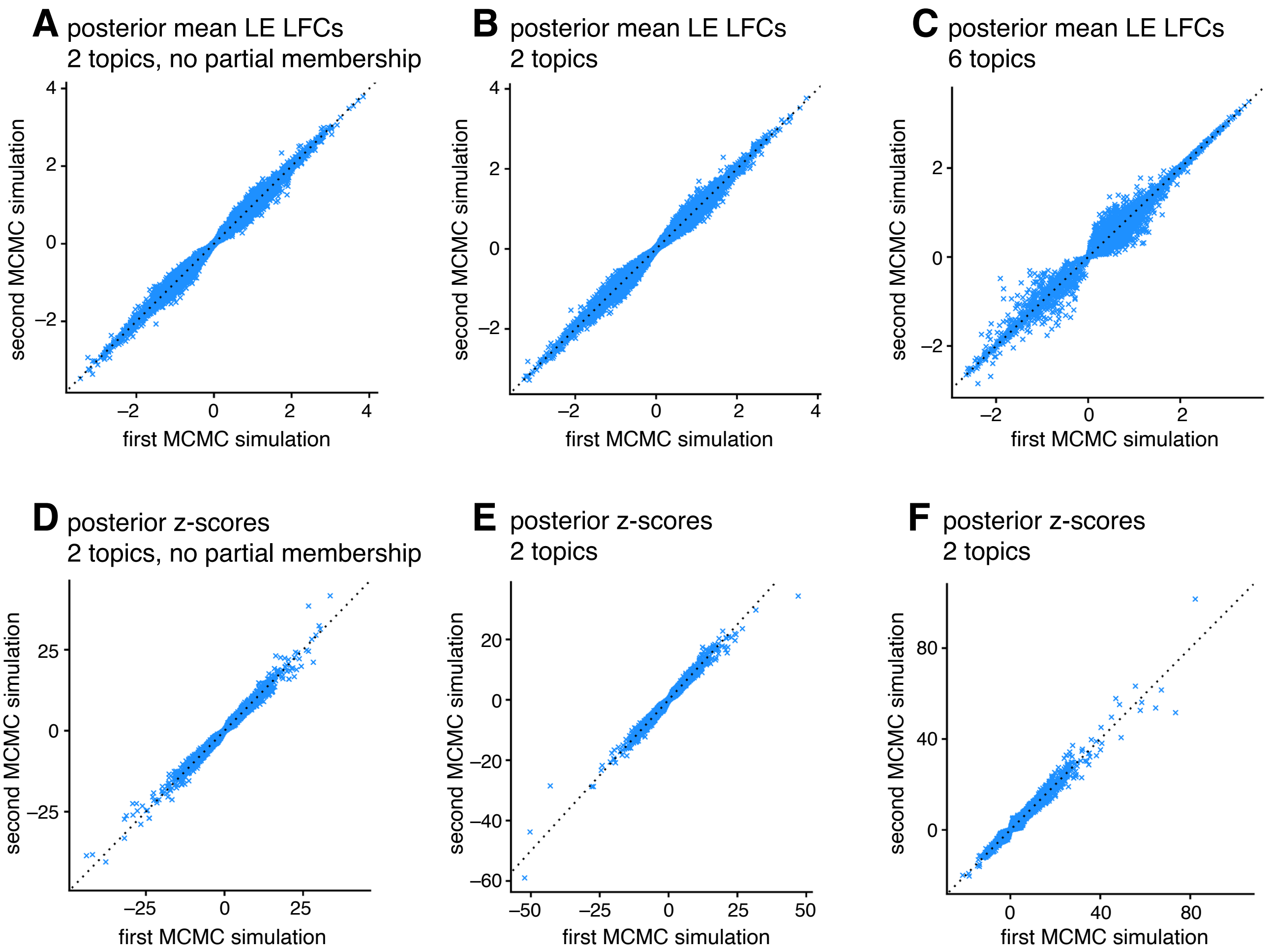

Fig. S6. Assessment of posterior computations for GoM DE analysis. MCMC is used to estimate posterior statistics, including posterior mean least extreme (LE) LFCs and posterior z-scores. To assess accuracy of the posterior calculations, we run the MCMC twice, and compare the posterior means and posterior z-scores after performing adaptive shrinkage. If the posterior mean LFCs and posterior z-scores are consistent between the two runs, this is a good indication that they are accurate. Panels A, B and C compare MCMC estimates of posterior mean least extreme LFCs, and Panels D, E and F compare MCMC estimates of posterior z-scores. In each scatterplot, 200,000 points are plotted for 10,000 genes $\times$ 20 simulated data sets: simulations with $K = 2$ topics, and without partial membership to these topics (Panels A, D); simulations with $K = 2$ topics with partial membership to these topics (B, E); simulations with $K = 6$ topics (C, F).

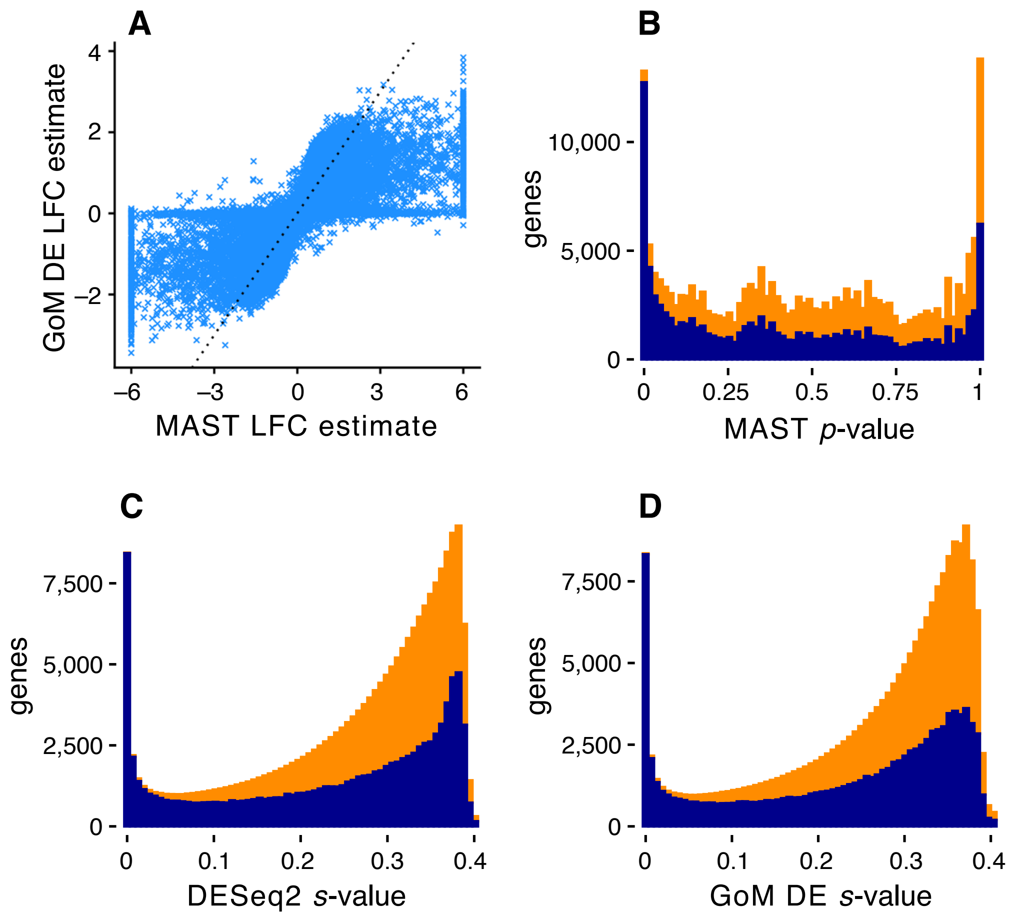

Fig. S7. Supplementary results from evaluating DE analysis methods in data sets in which cells are simulated *without* partial membership to topics. Panel A compares LFC estimates returned by MAST and the GoM DE analysis. In this plot, 200,000 points are plotted for the 10,000 genes × 20 simulated data sets. Panels B, C and D compare the distribution of *p*-values (MAST) and *s*-values (DESeq2, GoM DE), separately for true differences (dark blue) and non-differences (orange).

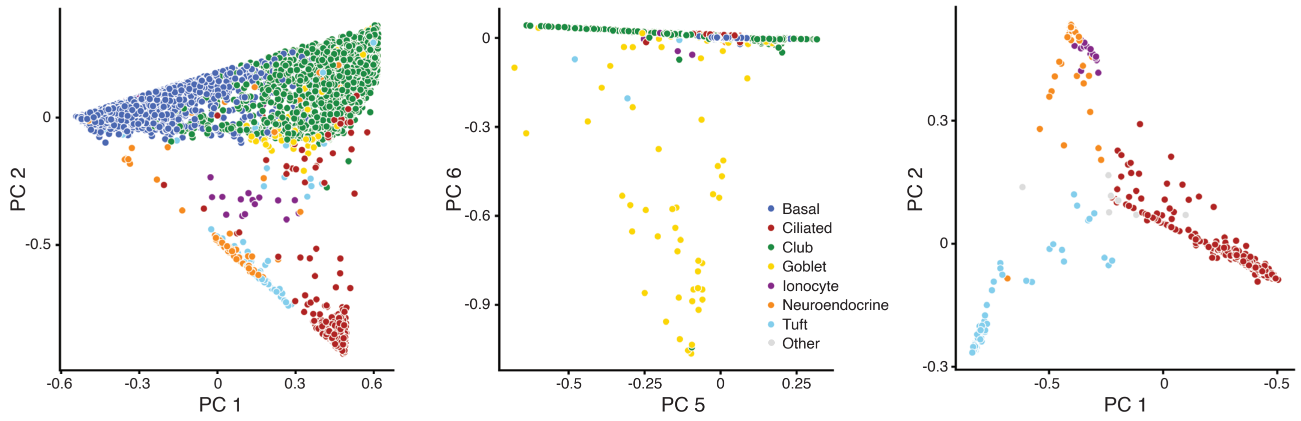

Fig. S8. *Left, middle:* Correspondence between the K = 7 topic model fit to epithelial airway data and the clustering from [1]. *Right:* Correspondence between the K = 5 topic model fit to the subset of rare epithelial cell types and the clustering from [1]. The plots show the 7,193 cells (left, middle) and 637 cells (right) projected onto principal components (PCs) of the estimated topic proportions **L**. The color of the points is varied according to the clustering obtained in [1] using a community detection algorithm. The “Other” label in the right-hand plot is for the small number cells included in the n = 637 subset of rare epithelial cell types that were labeled by [1] as belonging the basal, club and goblet clusters.

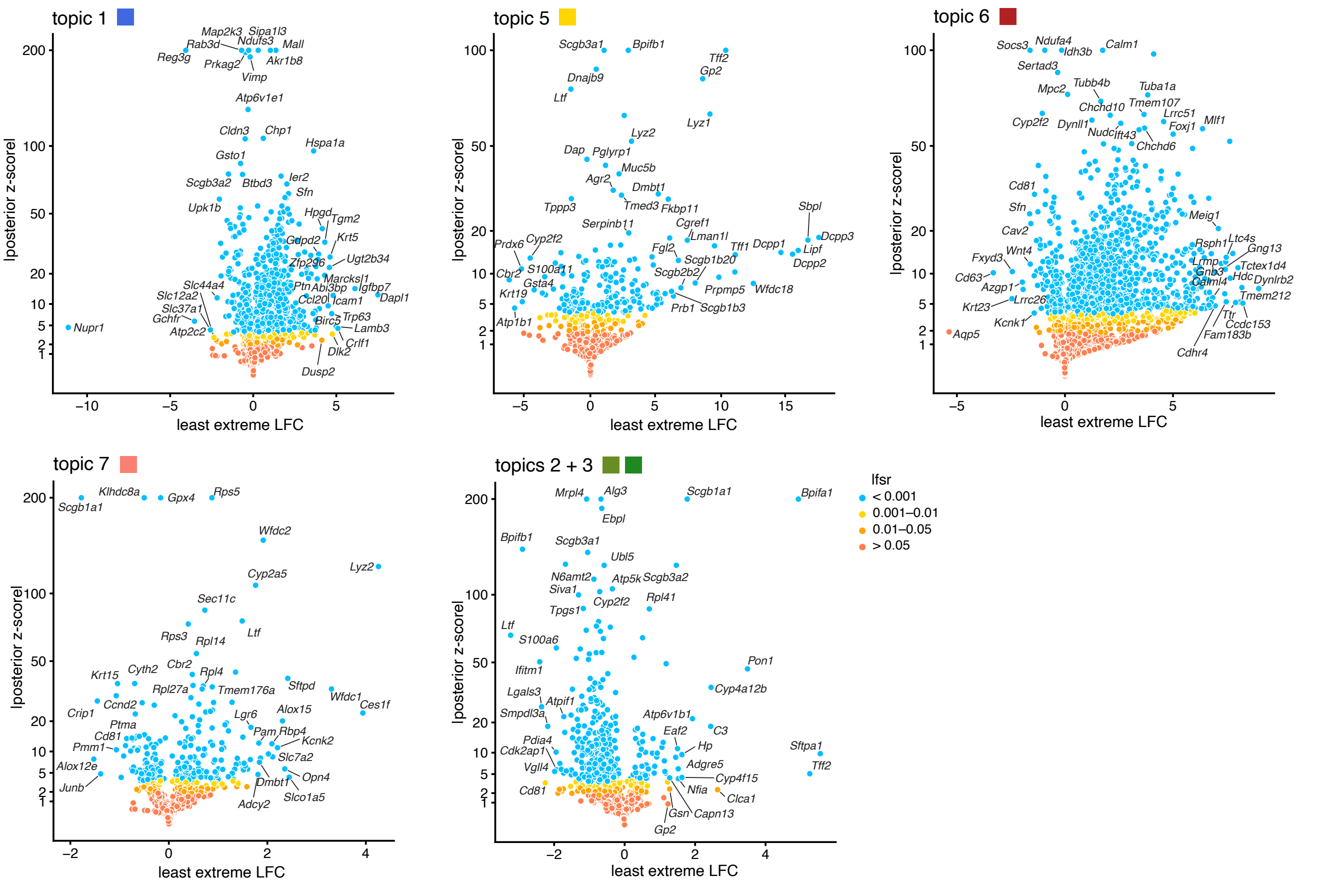

Fig. S9. GoM DE analysis of additional topics in $K = 7$ topic model fitted to epithelial airway data. The topic colors correspond to colors used in the Structure plot in Fig. 6A in the main text. The volcano plots show moderated posterior estimates of least extreme LFC vs. posterior z-score magnitudes for 18,388 genes. A small number of genes with extreme posterior z-scores are shown with smaller posterior z-scores so that they fit within the Y-axis range; actual posterior z-score statistics and other detailed statistics can be accessed in the interactive volcano plots provided in the supporting materials. For the GoM DE analysis of the combined topic, the topic proportions for the combined topic were defined as $l'_{i,2+3} = l_{i2} + l_{i3}$, then the GoM DE analysis proceeded using a modified topic proportions matrix $\mathbf{L}' \in \mathbf{R}^{18{,}388 \times 6}$, in which $l'_{ik} = l_{ik}$ for all $i, k = 1, 4\text{–}7$.

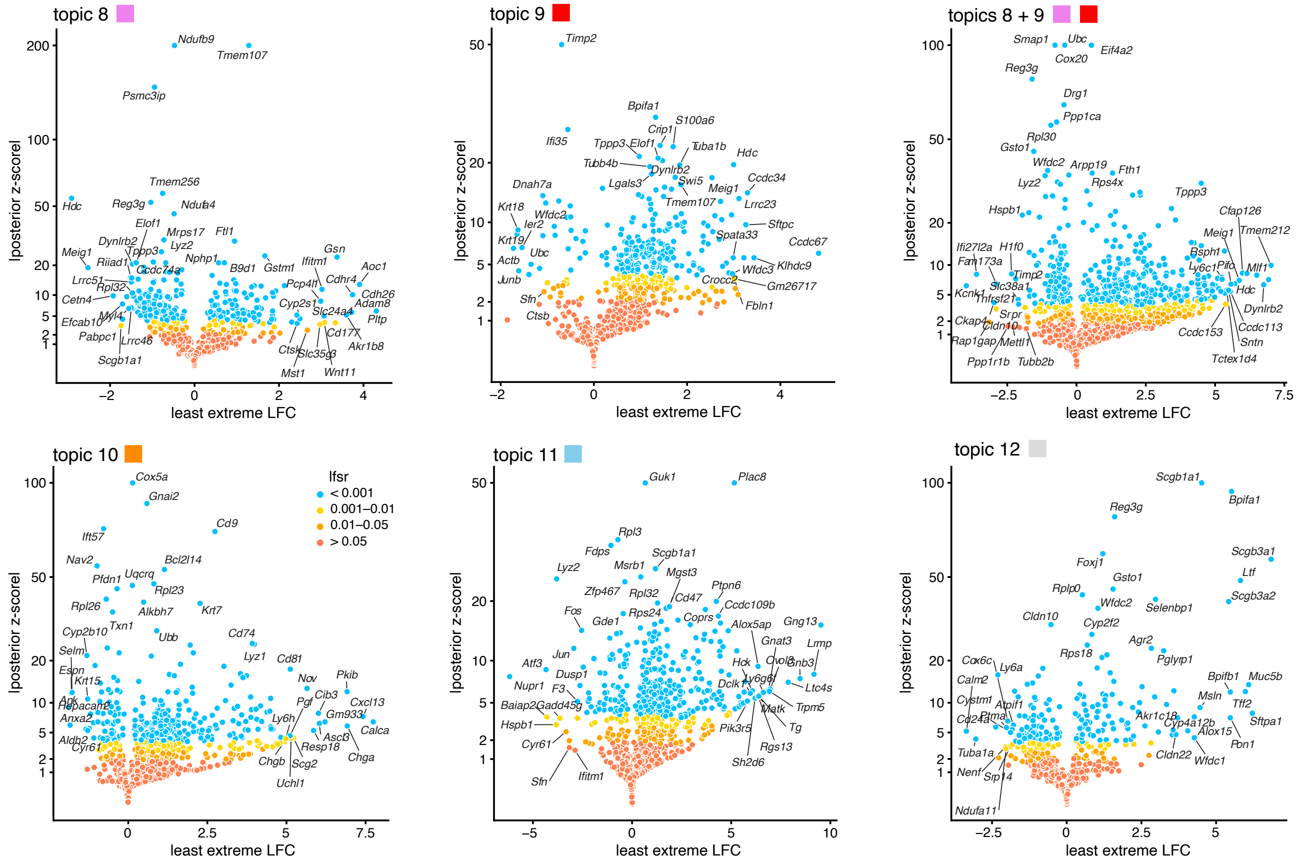

Fig. S10. GoM DE analysis of topics in the $K = 5$ topic model fitted to the rare epithelial cell type subset. The topic colors correspond to colors used in the Structure plot in Fig. 6A in the main text. The volcano plots show moderated posterior estimates of least extreme LFC vs. posterior *z*-score magnitudes for 15,594 genes. A small number of genes with extreme posterior *z*-scores are shown with smaller posterior *z*-scores so that they fit within the Y-axis range; actual posterior *z*-score statistics and other detailed statistics can be accessed in the interactive volcano plots provided in the supporting materials. For the GoM DE analysis of the combined topic, the topic proportions for the combined topic were defined as $l'_{i,8+9} = l_{i8} + l_{i9}$, then the GoM DE analysis proceeded using a modified topic proportions matrix $\mathbf{L}' \in \mathbf{R}^{15,594 \times 5}$, in which $l'_{ik} = l_{ik}$ for all $i, k = 10, 11, 12$.

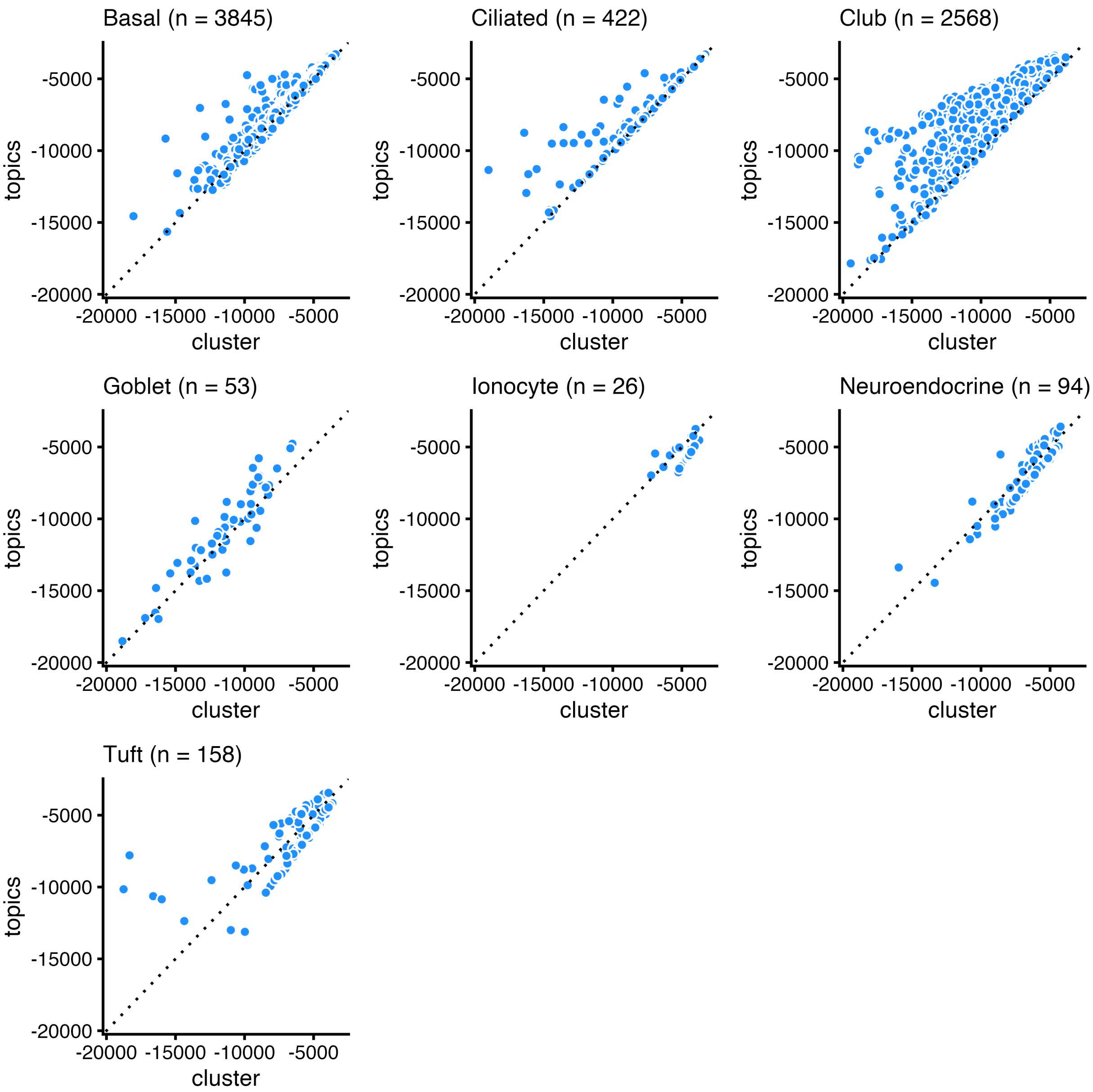

Fig. S11. Assessment of single-cell likelihoods for K = 7 topic model fit to epithelial airway data. In [1], the 7,193 cells were subdivided into 7 clusters corresponding to 7 cell types (basal, ciliated, club, goblet, ionocyte, neuroendocrine, tuft). Here we assess how well the topic model captures expression in these cell types by comparing topic model likelihoods against likelihoods under a simple multinomial model (eq. 1 in main text) in which all cells share the same underlying pattern of expression; that is, $\pi_{ij} = \pi_j$ for all cells i in the cluster. (The multinomial probabilities π_j in this simple model are estimated by maximum-likelihood; $\hat{\pi}_j = \sum_i x_{ij} / \sum_i s_i$, in which the sums are over all cells i assigned to the cluster.) The scatterplots compare, for each cell, the log-likelihood under this simple cluster-based multinomial model (X axis) against the topic model log-likelihood (Y axis). The topic model provides a better fit for cells above the diagonal (dotted line) and a worse fit for cells below the diagonal.

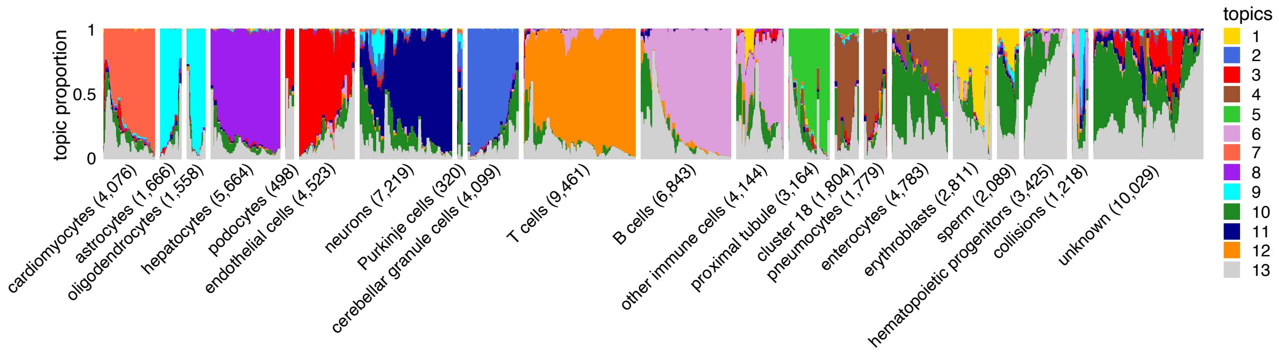

Fig. S12. Structure plot visualizing topic model fit to the Mouse Atlas sci-ATAC-seq data [2], with $K = 13$ topics. The cells are grouped by "cell type"—specifically the "cell_label" column of the metadata table which gives final cell-type assignments incorporating automated and manual assignments (see Table S1 in [2] for details). "Other immune cells" includes natural killer cells, monocytes, macrophages, microglia and dendritic cells. "Cluster 18" includes the cells—mostly kidney cells—labeled as "collecting duct", "loop of henle" and "DCT/CD". The numbers of parentheses next to each cell type label give the number of cells assigned to that cell type. Compare to Fig. 7A.

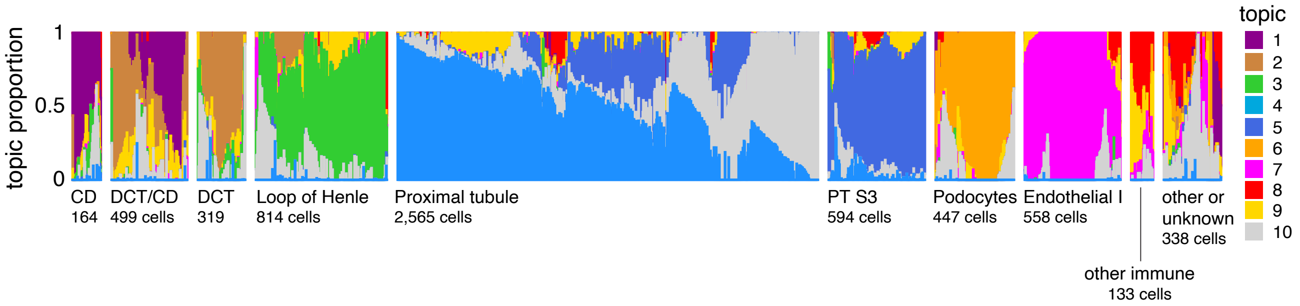

Fig. S13. Structure plots visualizing the topic model fit to the Mouse Atlas kidney cells, with $K = 10$ topics, in which the cells are arranged by the cell-type assignments estimated in [2]. Specifically, these are the final cell-type assignments incorporating automated and manual assignment; see also Table S1 in [2]. These cell-type assignments were retrieved from the "cell_label" column in the metadata file. The "other immune" label includes cells labeled as B cells, NK cells, T cells, dendritic cells, macrophages and monocytes.

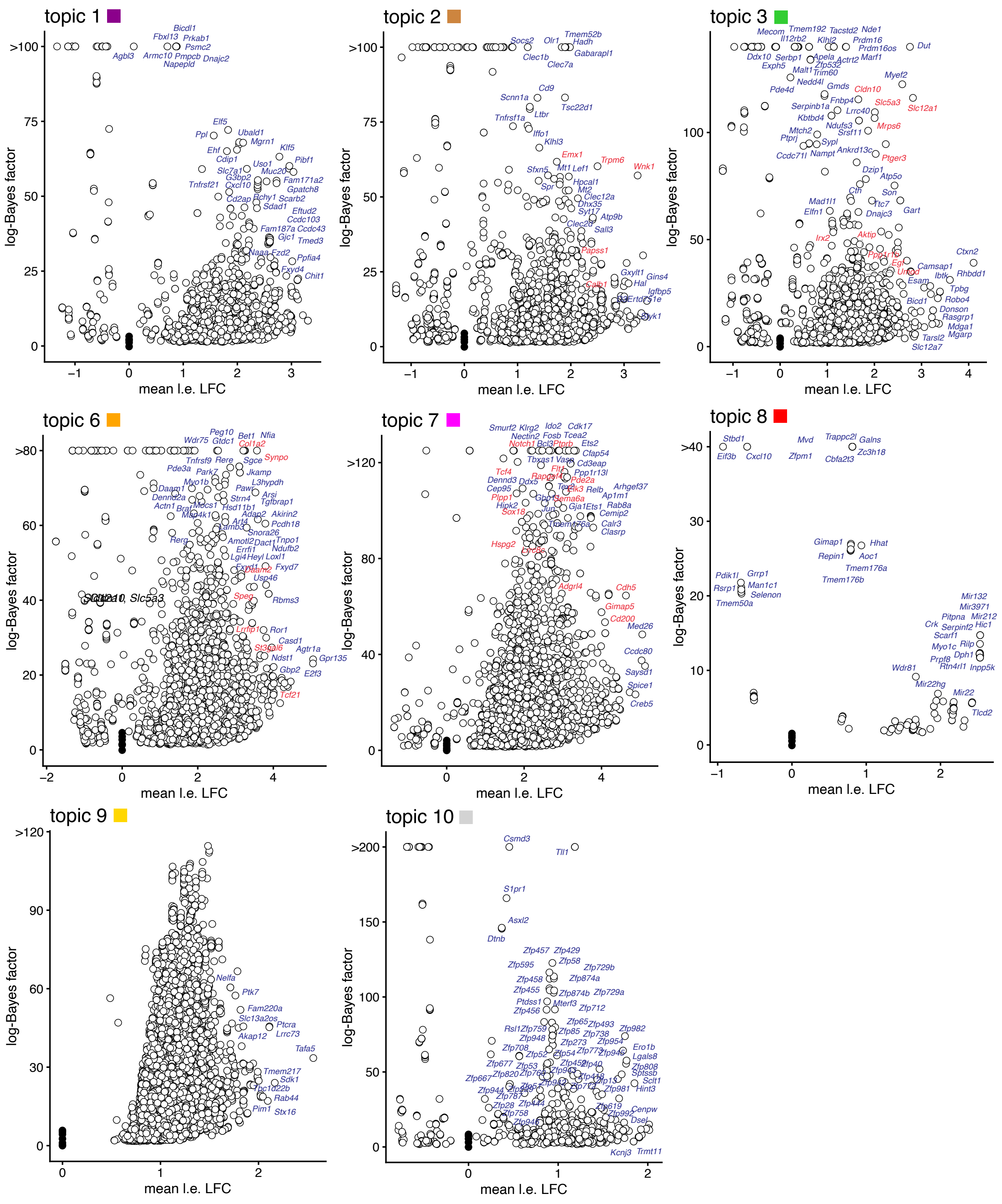

Fig. S14. Additional gene enrichment results for topics in Mouse sci-ATAC-seq Atlas kidney data based on differences in accessibility of peaks linked to genes by Cicero [3]. “Mean l.e. LFC” is the average least extreme LFC among all peaks connected to the gene, restricted to l.e. LFCs with *lfsr* < 0.05. Note that some genes at the top of the volcano plot may actually have a larger log-Bayes factor than what is shown; the log-Bayes factors were truncated to fit within the range of the volcano plot. Marker genes with cell-type-specific expression in distal convoluted tubule (topic 2), loop of Henle (topic 3), podocytes (topic 6) and endothelial cells (topic 7) are highlighted in red. These marker genes were identified from a differential expression analysis of single-cell RNA-seq profiles in healthy kidney cells [4] (see Table S1 of [4] for the full list of identified marker genes).

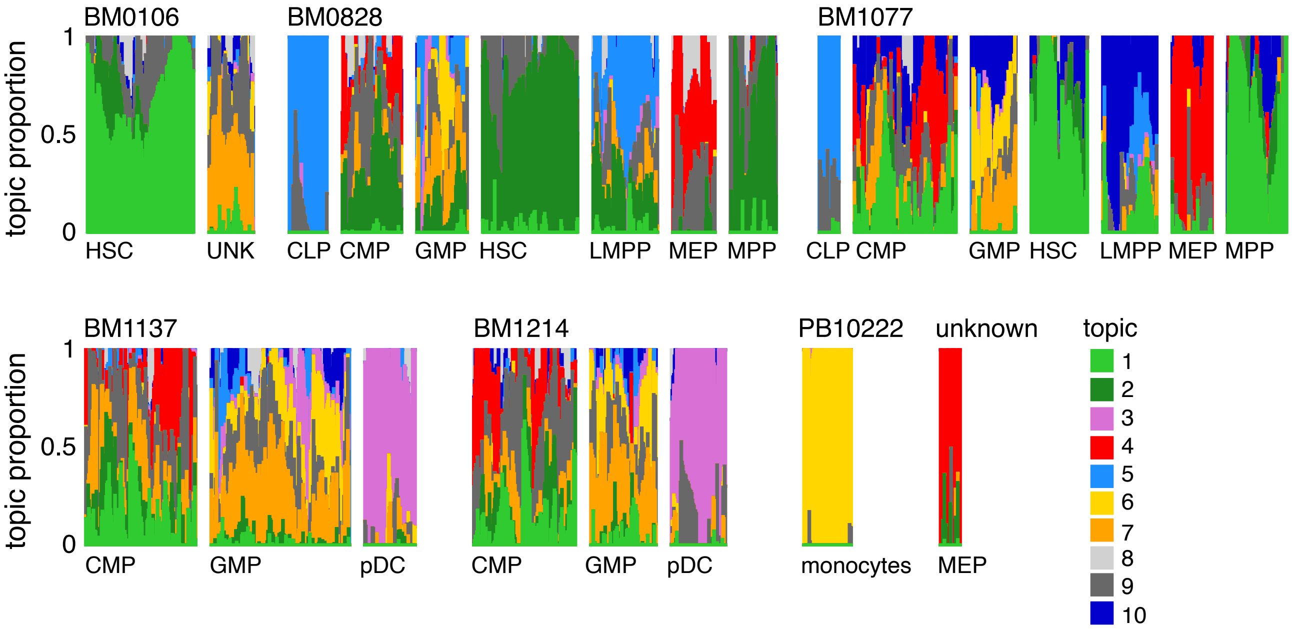

Fig. S15. Topic model shown in Fig. 8A in which the 2,034 cells are arranged by both donor ID and FACS cell type classification. Topic 2 predominantly occurs in donor BM0828, and topic 10 predominantly occurs in donor BM1077.

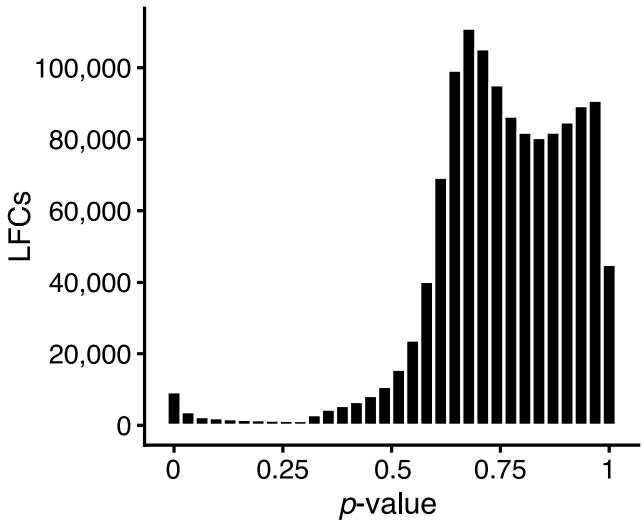

Fig. S16. Distribution of *p*-values for all topics from the GoM DE analysis of chromatin accessibility using the $K = 10$ topic model fitted to the hematopoietic system single-cell ATAC-seq data. Note there is one LFC estimate for each of the chromatin accessibility regions and for each topic. Also note that these *p*-values are not corrected for multiple testing (e.g., controlling for FDR).

References

[1] Montoro, D.T., Haber, A.L., Biton, M., Vinarsky, V., Lin, B., Birket, S.E., Yuan, F., Chen, S., Leung, H.M., Villoria, J., Rogel, N., Burgin, G., Tsankov, A.M., Waghray, A., Slyper, M., Waldman, J., Nguyen, L., Dionne, D., Rozenblatt-Rosen, O., Tata, P.R., Mou, H., Shivaraju, M., Bihler, H., Mense, M., Tearney, G.J., Rowe, S.M., Engelhardt, J.F., Regev, A., Rajagopal, J.: A revised airway epithelial hierarchy includes CFTR-expressing ionocytes. Nature **560**(7718), 319–324 (2018)

[2] Cusanovich, D.A., Hill, A.J., Aghamirzaie, D., Daza, R.M., Pliner, H.A., Berletch, J.B., Filippova, G.N., Huang, X., Christiansen, L., DeWitt, W.S., Lee, C., Regalado, S.G., Read, D.F., Steemers, F.J., Disteche, C.M., Trapnell, C., Shendure, J.: A single-cell atlas of *in vivo* mammalian chromatin accessibility. Cell **174**(5), 1309–1324 (2018)

[3] Pliner, H.A., Packer, J.S., McFaline-Figueroa, J.L., Cusanovich, D.A., Daza, R.M., Aghamirzaie, D., Srivatsan, S., Qiu, X., Jackson, D., Minkina, A., Adey, A.C., Steemers, F.J., Shendure, J., Trapnell, C.: Cicero predicts cis-regulatory DNA interactions from single-cell chromatin accessibility data. Molecular Cell **71**(5), 858–8718 (2018)

[4] Park, J., Shrestha, R., Qiu, C., Kondo, A., Huang, S., Werth, M., Li, M., Barasch, J., Suszták, K.: Single-cell transcriptomics of the mouse kidney reveals potential cellular targets of kidney disease. Science **360**(6390), 758–763 (2018)
