# Supplementary material for: Interpreting structure in sequence count data with differential expression analysis allowing for grades of membership
Source: bioRxiv. 2023 Mar 6:2023.03.03.531029. Preprint. [Version 1] doi: 10.1101/2023.03.03.531029 (PMC10028846; doi:10.1101/2023.03.03.531029)
Supplement: Supplementary file 3 [file media-3.zip › volcano_plots_montoro_droplet/volcano_plot_droplet_rare_tuft.html]

topic k1
